# Applied behavior analysis and the zoo: Forthman and Ogden (1992) thirty years later

**DOI:** 10.1002/jaba.969

**Published:** 2022-12-23

**Authors:** Eduardo J. Fernandez, Allison L. Martin

**Affiliations:** ^1^ School of Animal and Veterinary Sciences University of Adelaide; ^2^ Department of Psychological Science Kennesaw State University

**Keywords:** animal training, animal welfare, applied animal behavior, behavioral engineering, environmental enrichment, zoos

## Abstract

The field of applied behavior analysis has been directly involved in both research and applications of behavioral principles to improve the lives of captive zoo animals. Thirty years ago, Forthman and Ogden (1992) wrote one of the first papers documenting some of these efforts. Since that time, considerable work has been done using behavioral principles and procedures to guide zoo welfare efforts. The current paper reexamines and updates Forthman and Ogden's original points, with attention to the 5 categories they detailed: (a) promotion of species‐typical behavior, (b) reintroduction and repatriation of endangered species, (c) animal handling, (d) pest control, and (e) animal performances. In addition, we outline 3 current and future directions for behavior analytic endeavors: (a) experimental analyses of behavior and the zoo, (b) applied behavior analysis and the zoo, and (c) single‐case designs and the zoo. The goal is to provide a framework that can guide future behavioral research in zoos, as well as create applications based on these empirical evaluations.

Thirty years ago, Forthman and Ogden (1992) published one of the first papers to review the contributions of applied behavior analysis to zoos. Both authors were associated with the TECHLab (later, Georgia Tech Center for Conservation and Behavior), a partnership between Zoo Atlanta and the Georgia Institute of Technology led by Dr. Terry Maple (Maple, 2017). Dr. Maple pioneered, developed, and championed the concept of the *empirical zoo*, recognizing the potential of university–zoo collaborations to impact basic and applied research, animal management and welfare practices, and education (Fernandez, 2017; Lukas et al., 1998; Maple, 2007, 2008, 2017; Maple & Perdue, 2013). Dr. Maple, along with distinguished behavior analytic scholar Dr. M. Jackson (Jack) Marr, trained many researchers who went on to advance the application of behavior analytic principles in zoo settings (Maple, 2016, 2017, 2021; Maple & Segura, 2015).

At the time, single‐case designs to examine the effects of environmental enrichment were becoming an important cornerstone of zoo behavioral welfare research (Carlstead et al., 1991; Carlstead et al., 1993; Carlstead & Seidensticker, 1991; Newberry, 1995; Shepherdson et al., 1993; see later section on the use of single‐case designs in zoos). The concept of enrichment itself was largely derived from Markowitz's work on using operant conditioning to create desired behavioral changes in zoo animals, which was the focus of many of his publications in the late 1970s and 1980s (Markowitz, 1978, 1982; Markowitz & LaForse, 1987; Markowitz et al., 1978; for a review, see Fernandez & Martin, 2021). Thus, Markowitz's behavioral engineering practices, along with Forthman and Ogden's (1992) paper and the work of Dr. Terry Maple and the TECHLab, would help pave the way for this past 30 years of applied behavior analytic endeavors into behavioral welfare efforts in zoos.

The current review reexamines the topics introduced by Forthman and Ogden (1992) as ways in which applied behavior analysis can promote the conservation, education, entertainment, and welfare goals of modern zoos. Below, we address the five topics they discussed (i.e., promotion of species‐typical behavior, reintroduction and repatriation of endangered species, animal handling, pest control, and animal performances). The focus of each section is to provide updated examples of applied behavior analytic research and efforts that have occurred in each area since Forthman and Ogden's original publication. In addition, the review concludes with behavior analytic areas of interest and emphasizes current and future zoo‐based studies, including (a) experimental analyses of behavior and the zoo, (b) applied behavior analysis and the zoo, and (c) single‐case designs and the zoo. The primary goal is to provide both a historical and theoretical foundation to guide the future of applied behavior analytic work in zoos.

## Literature Review Criteria, Variables Coded, and Intercoder Agreement

**Figure 1:**
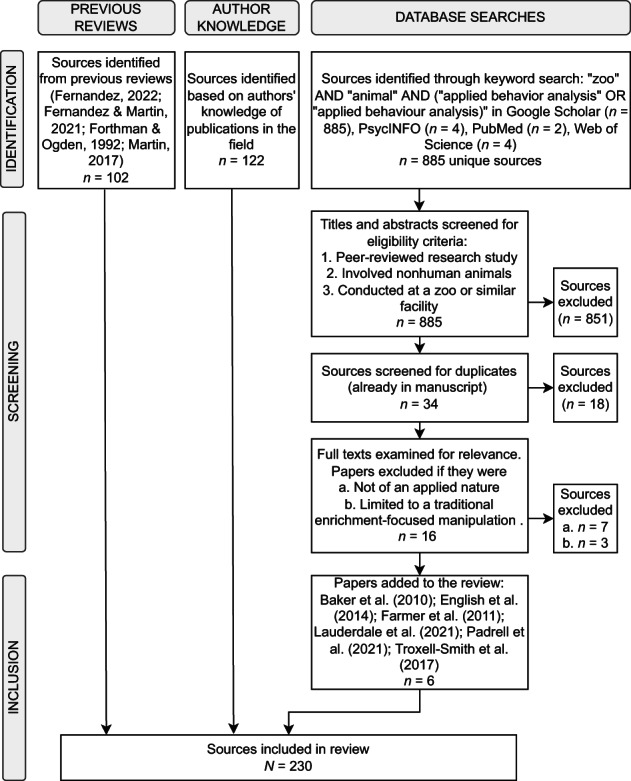
Flowchart of Literature Search

At its core, behavior analysis examines the relation between the environment and behavior. Thus, almost all zoo‐based behavioral studies can be viewed through a behavior analytic lens. Given the breadth of this topic, our goal was not to write a systematic review, but instead was to further the discussion begun by Forthman and Ogden (1992) about what the science of behavior analysis can contribute to the zoo. Nonetheless, to assist the reader in understanding our review process, we have provided additional information about our publication selection criteria. Our inclusion of literature was based largely on knowledge of publications within the field by both authors, including articles cited in prior literature reviews (Fernandez, 2022; Fernandez & Martin, 2021; Forthman & Ogden, 1992; Martin, 2017; 102 total papers) as well as literature familiar to us through the typical ways in which scholars stay current in their field (e.g., reading journals, conducting literature searches, citation alerts; 122 total papers). In addition, we conducted a supplemental Google Scholar search (July, 2022) and then additional searches via PsycINFO, PubMed, and Web of Science (September, 2022) based on the following criteria: “zoo” AND “animal” AND “applied behavior analysis” OR “applied behaviour analysis.” The Google Scholar search produced 885 results, and the other search engines produced eight results that overlapped with the Google Scholar findings. Based on titles and abstracts, we selected articles that: (a) were peer‐reviewed research studies, (b) involved nonhuman animals in some component of the research, and (c) were conducted at a zoo or similar facility that housed exotic animals. Based on these eligibility criteria, the search yielded 34 results. Articles were also removed if they were already included as one of the 224 papers identified in our preliminary review (18 papers removed). This exclusion criterion left 16 results. We then assessed the full text of articles to determine relevance for our review. We excluded papers if they were (a) not of an applied nature (observation‐only or otherwise not directly aimed at improving welfare; seven papers removed), or (b) limited to a traditional enrichment‐focused manipulation (not assessed for potential function; see Physical Variables section; three papers removed). Following the application of these exclusion criteria, six articles were added to the review, resulting in a total of 230 sources included in the review. Figure 1 details the results of our literature search.

Finally, given the large number of papers published in two emerging areas of research (functional analysis and preference assessments), we summarized these studies in tables (see Applied Behavior Analysis and the Zoo section later in the paper). This tabular summary required the categorization of articles on several dimensions. Exact agreement was used to assess intercoder agreement for functional analysis and preference assessment articles. Both authors independently coded 40% of the functional analysis articles for author and year (100% agreement), species (100% agreement), target behavior (100% agreement), experimental design (100% agreement), and primary function identified (100% agreement). Both authors also independently coded 32% of the preference assessment articles on author and year (100% agreement); species (100% agreement); type of stimuli used in each study (food, nonfood, symbols, or mixed/multiple; 90% agreement); and whether the study presented stimuli singly, in pairs, in arrays with three or more stimuli, or in mixed/multiple ways (90% agreement).

## Forthman and Ogden Revisited

### Promotion of Species‐Typical Behavior

For the better part of a century, zoos and zoo‐like facilities have been concerned with getting animals to behave similarly to their wild counterparts (Hediger, 1950, 1955; Morris, 1964; Yerkes, 1925). Early applied behavior analytic endeavors in zoos, such as the work of Markowitz and colleagues noted above (Markowitz 1978, 1982; Markowitz & LaForse, 1987; Markowitz et al., 1978), achieved some of these goals by developing simple operational definitions of the desired responses and using mechanical devices installed in exhibits to reinforce these contrived behaviors. However, these efforts were met with criticism, particularly with regard to how they related to nonnatural behaviors and nonnaturalistic environments (Hancocks, 1980; Hutchins et al., 1984). The eventual resolution would be an integrative approach that used both learning principles and understandings of species‐typical behaviors and settings to guide welfare‐based activities, such as environmental enrichment (Forthman‐Quick, 1984). Modern examples include using wild‐like enrichment activities and devices to encourage species‐typical foraging behaviors, such as foraging patches with Parma wallabies (*Macropus parma*) and Patagonian cavies (*Dolichotus patagonum*; Troxell‐Smith et al., 2017), as well as the use of artificial termite mounds with chimpanzees (*Pan troglodytes*; Padrell et al., 2021).

Although a detailed historical examination of the convergence of learning and evolution to understand behavior is beyond the scope of this paper, it is worth noting that the interest of Forthman and Ogden (1992) in species‐typical behavior from a learning perspective echoes concerns raised by Breland and Breland (1961), as well as Herrnstein (1977) when addressing the importance of attending to the evolutionary history of the organisms that are learning. Likewise, Skinner himself would make similar points about the importance of understanding the phylogeny of behavior to better understand how any response is selected (Morris et al., 2004; Skinner, 1966a; Skinner, 1984). In addition, behavior systems researchers emphasized nonlearned, biological components of behavior that would influence an organism's learning (Domjan, 1983; Shettleworth, 1993; Timberlake, 1993; Timberlake & Lucas, 1989), and more recently, behavior analysts such as Baum (2012) have echoed the importance of Phylogenetically Important Events (PIEs) on learned behavior. Finally, the extent to which researchers use species‐typical or “natural” behavior as an animal welfare metric across a variety of settings has been discussed and debated (Browning, 2020; Fraser, 2008; Hutchins, 2006). Nonetheless, species‐typical behavior serves as an important assessment and improvement welfare measure, particularly for the diversity of wild animals typically displayed in zoos.

Below, we consider two subcategories of promoting species‐typical behavior that Forthman and Ogden (1992) originally outlined: (a) physical variables, such as exhibit space, feeding schedules, and potential enrichment items; and (b) social variables, such as the way zoo animals are housed with other animals. For both subcategories, we focus on more recent behavioral advancements published after Forthman and Ogden, as well as how related research has been used in application to benefit the lives of zoo animals.

#### Physical Variables

Applied behavior analysis as a field is certainly familiar with understanding the importance of the environment on behavior, particularly as it relates to antecedents (e.g., discriminative stimuli and setting events) and consequences. One of the key physical variables studied in zoos and noted by Forthman and Ogden (1992) is environmental enrichment. Since the time of their original publication, hundreds of studies on enrichment in zoos have been published. Thus, an extensive examination of each of these studies is not possible within this review (see Shyne, 2006; Swaisgood & Shepherdson, 2005; Zhang et al., 2022 for a selection of meta‐analyses conducted on aspects of enrichment involving zoos or aquariums). However, a few noteworthy studies include examinations that have used common applied behavior analytic procedures to assess and enhance the welfare benefit of enrichment practices. For instance, preference assessments have been used to determine both the type and effectiveness of potential enrichment (Clayton & Shrock, 2020; Dorey et al., 2015; Fernandez et al., 2004; Fernandez & Timberlake, 2019b; Mehrkam & Dorey, 2014, 2015), and feeding schedules, including fixed‐ and variable‐time schedules, predictable versus unpredictable feeding schedules, and live prey feeding events have been used as forms of enrichment (Andrews & Ha, 2014; Bloomsmith & Lambeth, 1995; Fernandez, 2020; Fernandez, Myers, & Hawkes, 2021; Wagman et al., 2018). In all of the above, a core element, as stressed by Forthman and Ogden, is a functional evaluation of the physical variables of interest. This necessarily means using experimental manipulations, ideally those that include single‐case designs (e.g., reversal, multiple‐baseline designs; Alligood et al., 2017; Fernandez & Timberlake, 2008; Maple & Segura, 2015).

Another set of physical variables important for promoting desired species‐typical behaviors in zoo animals are the exhibits themselves. Following Forthman and Ogden's (1992) publication, a few zoo researchers and personnel have emphasized the importance of understanding how exhibit design influences the behavior and welfare of zoo animals, including the effects of using rotating exhibits (Coe, 2004), the effects of exhibit space use and choice (Owen et al., 2005; Ritzler et al., 2021), the effects of different exhibit structures and changes to exhibits (Carlstead et al., 1993; Fernandez et al., 2020), and the use of computer technology to modify exhibit interactions (Carter et al., 2021; Coe & Hoy, 2020). Again, like environmental enrichment, the emphasis here is on understanding the function of the physical exhibit variable on the behavior of the exhibited animal and for applied purposes. From a behavior analytic perspective, this is most readily accomplished through experimental analysis using single‐case designs (see Single‐Case Designs and the Zoo section below).

#### Social Variables

Forthman and Ogden (1992) stressed the importance of understanding social factors, including how animals in zoos are exhibited with other animals. Nonetheless, only a few studies have directly examined how changes in social structures, specifically changes in the number of individuals housed together, benefits the behavior of zoo animals. For example, several studies have explored changes in the number of elephants housed together on the behaviors of those elephants (Lasky et al., 2021; Schmid et al., 2001). Other researchers have examined differences in the social housing of black and gold howler monkeys (*Alouatta caraya*), including the reproductive success of monkeys under different social housing conditions (Farmer et al., 2011). Likewise, researchers have investigated the social dynamic of giraffes (*Giraffa camelopardalis*) under different management and housing conditions (Bashaw, 2011; Bashaw et al., 2007), including maternally raised or deprived giraffes (Siciliano‐Martina & Martina, 2018). One difficulty with these studies is that the changes in how animals are housed are not systematically manipulated. Zoos rarely have the luxury of engaging in such manipulations because they are both cost prohibitive and potentially detrimental to the welfare of their animals. However, a couple of studies have implemented systematic changes in the social housing of zoo animals, thus allowing for such experimental control. Rowden (2001) studied social interactions in Bulwer's wattled pheasants (*Lophura bulweri*) by changing the number of individuals housed together, either in pairs or larger groups. Similarly, Fernandez and Harvey (2021) used quasi‐experimental reversals to examine how changes in the social housing of African wild dogs (*Lycaon pictus*) impacted enclosure use. In both studies, these experimental manipulations allowed for greater prediction and control of the social variables of interest, which itself could allow for greater specificity in the social housing and management of zoo animals.

It is also worth noting that social variables can include the human–animal interactions observed in zoos, a concept often described as animal–visitor interactions (Davey, 2007; Fernandez et al., 2009; Hosey, 2000; Sherwen & Hemsworth, 2019). Historically, animal–visitor interactions were seen as problematic for the conservation education of visitors, as well as generally having a negative impact on the welfare of zoo animals (Kreger & Mench, 1995). However, in the past two‐plus decades, greater emphasis has been placed on the positive impact that visitors can have on zoo animals (i.e., visitor effects; Hosey, 2000) and that the zoo animals and the zoo itself can have on visitors (i.e., visitor experiences; Godinez & Fernandez, 2019; Learmonth et al., 2021). In addition, others have discussed human–animal interactions in zoos that do not involve visitors, including keepers or staff (Hosey et al., 2018; Ward & Melfi, 2015). The relevant factor is that behavior analysis offers a unique perspective for understanding all human–animal interactions observed in zoos, particularly as they relate to the directly observable behaviors offered by both animals and visitors. In addition, the use of experimental manipulations is necessary for distinguishing between visitor effects and visitor experiences. The assumption is often that changes observed in animal activity are the result of visitor presence; but, without proper experimental control, researchers cannot assume that visitors cause changes in animal activity or vice versa, or whether they are even causally related (Fernandez & Chiew, 2021).

### Reintroduction and Repatriation of Endangered Species

The conservation of species via reintroduction of animals born and reared in zoos has been a major goal of the modern zoo (Fa et al., 2011; Fernandez & Timberlake, 2008). Although many reintroduction efforts are carefully assessed, the assessments often do not involve quantitative data. Forthman and Ogden (1992) describe one species, the golden lion tamarin (*Leontopithecus rosalia*), for which empirical evaluations have been conducted to evaluate the effects of introducing captive‐bred zoo animals into the wild (Kleiman et al., 1986). Since 1992, these efforts have continued, including comparisons of wild‐born and captive‐born tamarins, examinations of semi‐free‐ranging populations in captivity, and the use of different types of environmental enrichment in captivity and in relation to species‐typical behaviors necessary for wild tamarins (Bryan et al., 2017; Castro et al., 1998; Price et al., 2012; Ruiz‐Miranda et al., 2019; Sanders & Fernandez, 2020; Stoinski et al., 1997, 2003). Other notable species examples include examinations of wild‐like captive conditions for the endangered black‐footed ferret (*Mustela nigripes*; Miller et al., 1998), the effects of releasing Oldfield mice (*Peromyscus polionotus subgriseus*) into testing settings meant to mimic the wild (McPhee, 2003), and captive breeding and rearing practices of Key Largo woodrats (*Neotoma floridana smalli*) to improve their successful reintroductions (Alligood et al., 2008, 2011; Wheaton et al., 2013). These empirical studies that focus on how the arrangement of environmental contingencies impact behavior and, as a result, the success of reintroductions, offer glimpses at how applied behavior analytic research efforts that focus on quantitative, experimental methods could help assess and improve reintroduction efforts for a variety of species found in zoos.

### Animal Handling

Forthman and Ogden (1992) detailed the importance of applied behavior analysis in improving the husbandry practices that are commonplace within zoos. As they noted, using behavioral principles, zoos have been able to move away from chemical or physical immobilization practices to conduct the routine veterinary care necessary for their animals. This in large part was a result of the work of Keller Breland and Marian Breland, who effectively demonstrated the use of positive reinforcement to increase voluntary participation of a wide variety of animal species in many diverse settings (for a review, see Fernandez & Martin, 2021).

Although the use of positive reinforcement to improve animal handling practices in zoos and similar exotic animal settings is now commonplace, only a handful of publications have empirically examined its effects. Denver Zoo personnel trained two species of antelope, nyala (*Tragelaphus angasi*) and bongo (*Tragelaphus eurycerus*), to voluntarily enter crates for blood draws and other veterinary procedures (Grandin et al., 1995; Phillips et al., 1998). Bloomsmith et al. (1998) and Veeder et al. (2009) successfully used reward‐based methods to train large groups of chimpanzees (*Pan troglodytes)* and mangabeys *(Cercocebus atys atys)* to move (i.e., shift) to different areas within their enclosures. Savastono et al. (2003) detailed reward‐based procedures to train a variety of behaviors for a dozen different zoo‐housed new world monkey species. Cheyenne Mountain Zoo personnel trained giraffes (*Giraffa camelopardalis reticulata*) to participate in radiographs and hoof care (Dadone et al., 2016). Researchers have also examined the potential enriching or behavioral welfare effects of training Asian elephants (*Elephas maximus*) to paint (English et al., 2014), as well as training rhesus macaques (*Macaca mulatta*) to present various body parts or emit simple responses on cue (e.g., sit or stand; Baker et al., 2010). Finally, Zoo Atlanta researchers documented the changes in both trainer and elephant (*Loxodonta africana africana*) behaviors (e.g., frequency of trainer‐delivered cues; latency of elephant compliance) during a transition to protected contact (i.e., no human enters the elephant's habitat), positive reinforcement‐based management system (Wilson et al., 2015).

All the above studies involved before/after comparisons of various reinforcement‐based procedures to increase targeted behaviors. More recently, several studies have directly examined the shaping process to train petting zoo sheep (*Ovis aries*), a petting zoo goat (*Capra hircus*), and an African crested porcupine (*Hystrix cristata*) for different desired behaviors, such as walking on a halter or touching and holding to a target (Fernandez, 2020; Fernandez & Dorey, 2021; Fernandez & Rosales‐Ruiz, 2021). In addition, Lauderdale et al. (2021) examined various features of training sessions on bottlenose dolphins' (*Tursiops truncatus*) beaching/shifting responses, including number of sessions and trials, mean trials per session, time between sessions, criteria changes between trials, and magnitude of reinforcers delivered. Shaping research focused on continuous learning achievements, rather than pre‐ posttest training results, should facilitate our understanding of the conditions that are more likely to improve zoo animal handling practices. Regardless, the idea is simple: Positive reinforcement‐based animal handling and training practices can increase the likelihood of better physiological welfare for zoo animals, in addition to potentially functioning as behavioral enrichment (Fernandez, 2022).

### Pest Control

Forthman and Ogden (1992) proposed that applied behavior analytic techniques could be useful in managing free ranging “pest” species such as rodents and birds that enter zoo exhibits and pose potential for disease transmission and other hazards. Although this area has not seen a large growth in behavior analytic research, the potential remains. Before implementing a pest‐management program, zoo personnel must take the behavior of both pest species and zoo‐housed species into account. Applied behavior analysis could offer guidance in this area. Most pest control techniques focus on antecedents that attract or repel animals, and preference assessments can be useful in determining traps an animal is likely to enter (Carey et al., 1997) or baits an animal is likely to consume (Allsop et al., 2017; Morgan, 1990). In addition, if a potentially harmful bait will be used in a zoo enclosure, preference assessments and/or conditioned taste aversion can help to ensure that the bait is unlikely to be consumed by nontarget animals (Clapperton et al., 2015, 2014). Taking a more natural approach, antecedent manipulations could be used to attract natural predators such as barn owls to zoo grounds to help reduce nuisance rodent populations (Antkowiak & Hayes, 2004). The presumption is that the increased natural predators themselves would impose no direct threat to the exhibited zoo animals, which itself is an empirical question worthy of applied behavior analytic study.

### Animal Performances

The training of marine mammals for shows played a key role in the promotion of regular husbandry training procedures in zoos, which has been essential as an application for improving zoo animal lives. As noted earlier, this was due in large part to the influence of Keller and Marian Breland in bringing behavior analytic principles to a variety of settings, which included show animals located in dolphinariums (for a review, see Fernandez & Martin, 2021). Since then, the use of positive reinforcement to promote voluntary involvement in husbandry practices and improve behavioral welfare has been a hallmark for show animals (Brando, 2012; Eskelinen et al., 2015). Nonetheless, in recent years greater concern has been placed on the use of animals for shows or performance, including whether animals such as cetaceans (whales and dolphins) should exist in any form of captivity (Rose & Parsons, 2019). It is also worth noting that modern zoos have updated their focus on animal performances to include educational efforts that promote the conservation and welfare of the individuals and species involved in such shows (D'Cruze et al., 2019). In the past 30 years, the public and the zoos themselves have had a dramatic change in the perceptions of the purpose of animal performances, including the outcomes of these activities on the well‐being of those animals.

Although Forthman and Ogden (1992) suggested that animal performances improve the physiological and psychological welfare of animals, it is only more recently that studies have investigated the behavioral effects of animal performances or similar performance‐like interactions on the welfare of those animals. For instance, Kyngdon et al. (2003) found that short‐beaked common dolphins (*Delphinus delphis*) trained to engage in a Swim‐with‐Dolphins program increased their surfacing and use of outside areas during programs, but otherwise showed few behavioral changes before, during, or after the interactions (thus suggesting little to no negative welfare impact from such programs). Similarly, Trone et al. (2005) found few behavioral differences for bottlenose dolphins (*Tursiops truncatus*) in the times before or after interaction programs. They additionally noted an increase in play behaviors following interactions, which may have indicated potential positive welfare effects of the interactions. Finally, Fernandez, Upchurch, and Hawkes (2021) examined the effects of a visitor feeding program on the activity of the exhibited elephants. They successfully demonstrated that the feeding programs increased overall activity and decreased undesired behaviors, such as stereotypies. Thus, as the purpose of animal performances in zoos has changed, this should require greater understanding of the impact of such shows on the animals. Future applied behavioral research in zoos could focus more directly on some of these direct welfare impacts, as well as what visitors learn from such shows.

## Current and Future Directions

Below, we consider additional areas of focus that are of mutual interest to both the field of behavior analysis and zoos. These three foci include: (a) experimental analyses of behavior and the zoo, (b) applied behavior analysis and the zoo, and (c) single‐case designs and the zoo. We consider each of these areas because basic and applied research have been traditional distinctions for the field of behavior analysis, and each focus has benefits they could bring to future zoo research and application through some of their areas of interest. Likewise, single‐case designs have been a staple of behavior analytic research and practice, and greater use of such methods could directly benefit the welfare of zoo animals.

### Experimental Analyses of Behavior and the Zoo

Although a detailed examination of all the potential contributions of the experimental analysis of behavior to zoo research exceeds the purpose of this review, it is worth pointing out several areas of overlap that are of mutual interest to both zoos and basic research in behavior analysis. These three areas include studies that involve (a) contrafreeloading, (b) autoshaping, and (c) conditioned reinforcement. Below we focus on how experimental studies of these topics could simultaneously improve our scientific understanding of behavior while contributing to behavioral welfare improvements or other applications meant to improve the lives of zoo animals.

#### Contrafreeloading

*Contrafreeloading* describes the phenomenon where animals will choose to work for food (e.g., press a lever or operate similar operanda) over freely available food (Jensen, 1963; Neuringer, 1969). Although the concept of contrafreeloading has been investigated across multiple species and settings (for a review, see Inglis et al., 1997), only a few studies have examined contrafreeloading in zoos. For instance, McGowan et al. (2010) demonstrated that captive grizzly bears (*Ursus arctos horribilis*) would spend at least a portion of their time retrieving food from ice blocks or enrichment boxes over consuming free food alone. Vasconcellos et al. (2012) showed that captive maned wolves (*Chrysocyon brachyurus*) would spend more time searching for food scattered across vegetation, as well as consume half their diet from scattered feedings when compared to food delivered on a tray in one section of their enclosures. Sasson‐Yenor and Powell (2019) demonstrated that several zoo‐housed giraffes (*Giraffa camelopardalis*) were more likely to contrafreeload when presented simultaneously with easily accessed or more time‐consuming enrichment foraging devices.

The topic of contrafreeloading itself raises interesting theoretical questions about the interplay between learning and evolved foraging patterns (Killeen, 2019; Timberlake, 1984). In addition, contrafreeloading should be of great interest to zoos, because many of the problem behaviors observed in exhibited animals (e.g., stereotypies) have been associated with species‐typical foraging patterns (Fernandez, 2021; Fernandez & Timberlake, 2019a). It could be argued that all food‐based enrichment deliveries elicit or set the occasion for behavior like that observed in contrafreeloading procedures, and therefore should equally be of interest for testing behavioral theories of contrafreeloading while benefiting exhibited animals by increasing species‐typical foraging patterns and increasing overall activity.

#### Autoshaping

*Autoshaping* describes a phenomenon in which voluntary behavior, such as key pecking, is respondently conditioned (Brown & Jenkins, 1968; Williams & Williams, 1969). Since the time of its discovery, it has been the focus of intense theoretical scrutiny, including the extent to which operant contingencies alone can successfully describe the behaviors observed under standard laboratory conditions (Timberlake, 2004; Timberlake & Lucas, 1989). The concept of autoshaping itself should be of interest to both basic researchers and practitioners when examining their effects on zoo animals. For example, researchers have been able to use respondent conditioning procedures to increase the reproductive success of animals (Domjan et al., 1998; Gaalema, 2013; Hollis et al., 1997). Given that reproductive behavior is both a voluntary activity and of great applied interest for zoo animals on Species Survival Plans (SSP; AZA, 2022), behavior analysts would do well to help foster empirical studies done on autoshaping and reproduction in zoos. Likewise, autoshaping has been used as a training procedure to increase time spent contacting a pool‐based enrichment feeding device, and therefore time spent swimming, in zoo penguins (Fernandez et al., 2019). Again, like efforts using autoshaping to increase reproductive efforts, autoshaping could be studied across a plethora of species in zoos, while simultaneously increasing enrichment device interactions, and thus have applied welfare benefits in the process of conducting such theoretical examinations.

#### Conditioned Reinforcement

*Conditioned reinforcement*, whereby a secondary stimulus associated with a primary reinforcer comes to maintain operant behavior, has been an important concept for the experimental analysis of behavior (Lattal & Fernandez, 2022; Pierce & Cheney, 2013). As early as 1938, Skinner described how a clicking sound initially paired with food reinforced lever pressing in the absence of food deliveries. Both Skinner (1951) and Breland and Breland (1951) would later describe the importance of using conditioned reinforcement to train animals outside of the lab. The idea was popularized by Pryor (1984) as a form of “clicker training” that could be used to train dogs, as well as other animals, for a variety of applied purposes. In the lab, the concept would become a focal point for understanding operant procedures (for reviews, see Fantino & Romanovich, 2007; Kelleher & Gollub, 1962). Similarly, the use of conditioned reinforcement for applied purposes has been examined, including whether conditioned reinforcement successfully improves some dimension of responding, such as speed of acquisition (Chiandetti et al., 2016; Dorey et al., 2020; Dorey & Cox, 2018; Gilchrist et al., 2021; Pfaller‐Sadovsky et al., 2020).

Although the importance of understanding conditioned reinforcement for both basic and applied research purposes remains, a divide still exists between information obtained from the lab and the field. For instance, early confusion existed on delivering conditioned reinforcers in the absence of primary reinforcers, an idea originally thought of as a form of intermittent reinforcement (often incorrectly described as “variable reinforcement”; Fernandez, 2001). Likewise, laboratory research on the importance of delay‐reduction and information hypotheses of conditioned reinforcement (each relating to the ability of some stimulus to successfully predict the occurrence of a following primary reinforcer more rapidly or accurately, respectively) have rarely been acknowledged outside of the lab, or only loosely identified or contrasted to marking/bridging hypotheses as they might apply in and outside of the lab (Dorey & Cox, 2018; Egger & Miller, 1962; Fantino, 1969; Williams, 1994). Zoos could provide a testing ground for translational research that experimentally examines the theoretical underpinnings of conditioned reinforcement, while providing a better connection between lab and field studies of conditioned reinforcement and improving the training procedures vital to the welfare of zoo animals.

### Applied Behavior Analysis and the Zoo

In addition to using behavioral science to optimize existing animal management practices such as environmental enrichment, researchers and zoo personnel have also borrowed specific techniques and protocols from applied behavior analytic clinical practices and modified them for use in applied animal settings. This represents a unique reverse‐translational cycle (Dixon et al., 2016; Edwards & Poling, 2011; Gray & Diller, 2017), where (a) basic principles from animal laboratory studies are used to (b) develop effective behavioral assessments and treatments in human clinical settings, and then (c) these human clinical protocols are used to improve captive animal care and welfare. As pointed out by others (Bloomsmith et al., 2007; Gray & Diller, 2017; Maple & Segura, 2015; Martin, 2017), the research and clinical approaches of behavior analysts have commonalities with the needs of zoos that make the transfer of behavioral technologies between the two settings beneficial. These commonalities include treatments developed for and evaluated at the individual level (Alligood et al., 2017; DeRosa et al., 2021; Fisher, Groff, & Roane, 2021; Saudargas & Drummer, 1996) and a focus on both building skills (Fisher, Piazza, & Roane, 2021; or, in zoos, promoting species‐typical behaviors) and decreasing behavioral excesses, including some of the same topographies of problem behavior seen in both settings (Bloomsmith et al., 2007). Below, we focus on two areas in which methods developed in applied behavior analytic clinical practice have been used in animal settings: (a) functional analysis, and (b) preference assessments, as well as on (c) untapped applications of applied behavior analytic protocols.

#### Functional Analysis

One example of this reverse translational research is the functional analysis technique. Drawing from his work with laboratory animals, Skinner (1953) developed the conceptual foundation for the functional analysis, emphasizing the impact of environmental events on behavior. Iwata et al. (1982/1994) then formalized a behavioral protocol that identified the existing environmental contingencies that reinforce and maintain problem behaviors. Over the past four decades, behavior analysts have successfully used function‐based treatments in human clinical settings to reduce behaviors including self‐injury, aggression, disruptiveness, and food refusal (Beavers et al., 2013; Hanley et al., 2003). More recently, researchers have used this same approach to successfully assess and treat problem behaviors in captive animals, including in zoo‐housed species. Functional analysis has been used to assess and treat self‐directed behaviors as well as disruptive or aggressive behaviors in nonhuman primates and in birds (Table 1). In all cases, a function‐based treatment consisting of some combination of extinction, differential reinforcement, noncontingent reinforcement, or some combination, have successfully reduced these problem behaviors (Dorey et al., 2009; Farmer‐Dougan, 2014; Franklin et al., 2022; Martin et al., 2011; Morris & Slocum, 2019).

**Table 1:** Summary of Functional Analysis Studies Involving Zoo‐Housed Species

Author(s) (Year)	Species	Target Behavior(s)	Experimental Design	Primary Function*
Dorey et al. (2009)	Olive baboon (*Papio hamadryas anubis*)	Self‐directed behavior (hair pulling, hand biting, foot biting)	Multielement	Positive reinforcement (attention from humans)
Farmer‐Dougan (2014)	Black‐and‐white ruffed lemur (*Varecia variegate variegata*)	Disruptive/aggressive behavior (aggression toward humans)	Other/unable to determine	Other/unable to determine
Franklin et al. (2022)	Rhesus macaque (*Macaca mulatta*)	Disruptive/aggressive behavior (noise)	Multielement	Positive reinforcement (food)
Martin et al. (2011)	Chimpanzee (*Pan troglodytes*)	Disruptive/aggressive behavior (human‐directed feces throwing, object throwing, and spitting; screaming, cage shaking)	Reversal	Positive reinforcement (attention and juice from humans)
Morris & Slocum (2019)	Black vulture (*Coragyps atratus*)	Self‐directed (feather‐plucking)	Multielement	Positive reinforcement (attention from humans)

* Primary function is the function identified as having the highest levels of behavior.

This functional approach to reducing abnormal behaviors exhibited by animals has many benefits, but it also has some limitations. Although most of the functional analyses conducted with zoo‐housed species have involved primates (Table 1), the inclusion of a vulture (Morris & Slocum, 2019) as well as the success of this approach in companion dogs (*Canis lupus familiaris*) and cats (*Felis catus*; Dorey et al., 2012; Hall et al., 2015; Mehrkam et al., 2020; Pfaller‐Sadovsky et al., 2019; Salmeron et al., 2021; Winslow et al., 2018) suggests that it can be useful across a range of species. However, these assessments are time‐ and labor‐intensive, and they can only be used to assess antecedents and consequences that can be systematically presented and withdrawn. Indeed, most functional analyses of zoo‐housed species identified either attention (i.e., attention function), human‐delivered food items (i.e., tangible function), or both, as the reinforcer for the problem behaviors (Table 1). Thus, in zoo settings, this approach seems especially useful to assess problem behaviors that may be maintained by interactions with zookeepers or visitors. However, many abnormal behaviors in zoo‐housed animals are likely to be maintained by consequences from other animals or by nonsocial (or “automatic”) reinforcers such as sensory stimulation or a decrease in arousal. These behaviors are more challenging to assess and treat; however, the behavior analytic literature can offer some guidance in these areas as well, including possibilities like antecedent assessments, noncontingent reinforcement, sensory extinction, or sensory‐matched enrichment (see Martin, 2017).

#### Preference Assessments

Another behavioral protocol developed in human clinical settings that has been of use in zoos is the stimulus or reinforcer preference assessment. In applied behavior analytic work with children, empirical preference assessments are conducted in educational settings, clinics, and hospitals to determine items or activities that are likely to serve as positive reinforcers for desired behaviors (Saini et al., 2021). Protocols include those in which items are presented one at a time (Pace et al., 1985), in pairs (Fisher et al., 1992), or in an array with multiple stimuli (DeLeon & Iwata, 1996), and selection is measured. Items ranked higher in preference (i.e., those that were selected most) using these methods were often found to function as more effective reinforcers for various desired behaviors than lower preference items (e.g., Carr et al., 2000; Lee et al., 2010; Pace et al., 1985; Piazza et al., 1996). Given the wide use of positive reinforcement training in zoos (Fernandez & Martin, 2021), researchers and zoo personnel recognize the importance of using highly preferred items to make training as efficient as possible. In addition, foods chosen by human or animal caregivers have been found to have low correlation with preferences determined empirically (e.g., Cote et al., 2007; Gaalema et al., 2011; Green et al., 1988; Mehrkam & Dorey, 2015), emphasizing the need for empirical assessments.

The three decades since Forthman and Ogden's (1992) paper have seen a surge in the use of empirical preference assessments in zoo‐housed species. As summarized in Table 2, researchers have empirically determined preferences for a wide variety of species, from invertebrates to apes. Most preference assessments have involved food, but some have involved other stimuli, including enrichment items, scents, and activities (see Table 2). Most studies have used some variation of free‐operant or paired‐choice presentations, but methods involving single‐item presentations and arrays with three or more stimuli have also been used (see Table 2). Additionally, there have been some novel research extensions in that some researchers have used symbolic representations of items (e.g., images, tokens) to facilitate choice whereas others have adapted methods by presenting stimuli to groups of animals rather than single animals (see Table 2).

**Table 2:** Summary of Preference Assessment Studies Involving Zoo‐Housed Species

Author(s) (Year)	Species	Stimuli	Presentation
Addessi (2008)	Capuchin monkeys (*Cebus apella*)	Food	Paired
Addessi et al. (2005)	Capuchin monkeys (*Cebus apella*)	Food	Paired
Addessi et al. (2010)	Capuchin monkeys (*Cebus apella*)	Mixed/multiple (food, symbols/tokens representing foods)	Mixed/multiple (paired, array)
Bloomfield et al. (2015)	Sumatran orangutans (*Pongo pygmaeus abelii*), Bornean (*Pongo pygmaeus pygmaeus)* and Sumatran orangutan hybrid	Nonfood (views of humans)	Single
Brox et al. (2021)	Slender‐tailed meerkats (*Suricata suricatta*)	Food	Paired (presented to groups)
Clay et al. (2009)	Sumatran organutans (*Pongo pygmaeus abelii*); Bornean orangutan (*Pongo pygmaeus pygmaeus*)	Food	Paired
Clayton & Shrock (2020)	Bengal tigers (*Panthera tigris tigris*); Siberian (*Panthera tigris altaica*) and Bengal tiger hybrid	Mixed/multiple (scents; food‐based enrichment; nonfood enrichment)	Mixed/multiple (Paired, Array)
Cox et al. (1996)	California sea lion (*Zalophus californianus*)	Symbols (drawings representing foods)	Paired
Dixon et al. (2016)	Madagascar hissing cockroach (*Gromphordahina portentosa*)	Food	Array
Dorey et al. (2015)	Gray wolves (*Canis Lupus)* and Artic wolves *(Canis Lupus Arctos*)	Mixed/multiple (food‐based enrichment; symbols / items representing training activities)	Paired
Fay & Miller (2015)	Rothschild giraffes (*Giraffa camelopardalis rothschildi*)	Nonfood (scents)	Paired
Fernandez & Timberlake (2019b)	Ring‐tailed lemurs (*Lemur catta*), red ruffed lemurs (*Varecia rubra*), collared lemurs (*Eulemur collairs*), and blue‐eyed black lemurs (*Eulemur flavifrons*)	Food	Paired
Fernandez et al. (2004)	Cotton‐top tamarins (*Saguinus oedipus*)	Food	Paired
Gaalema et al. (2011)	Giant pandas (*Ailuropoda melanoleuca*) and African elephants (*Loxodonta africana*)	Food	Paired
Hopper et al. (2019)	Gorilla (*Gorilla gorilla gorilla*)	Mixed/multiple (food, symbol/images of food)	Paired
Huskisson et al. (2020)	Gorillas (*Gorilla gorilla gorilla*), chimpanzees (*Pan troglodytes*), Japanese macaques (*Macaca fuscata*)	Symbols (images of food)	Paired
Huskisson et al. (2021)	Western lowland gorillas (*Gorilla gorilla gorilla*), chimpanzees (*Pan troglodytes*), Japanese macaques (*Macaca fuscata*)	Symbols (images of food, computer icon representing a random food)	Paired
Martin et al. (2018)	Rhesus macaques (*Macaca mulatta*)	Food	Array
Mehrkam & Dorey (2014)	Galapagos tortoises (*Chelonoidis nigra*)	Mixed/multiple (non‐food/enrichment, symbol/items representing human interaction activities)	Paired
Mehrkam & Dorey (2015)	Squirrel monkeys (*Saimiri*); springbok (*Antidorcas marsupialis*), red‐billed hornbirds (*Tockus erythrorhynchus*), Eastern indigo snake (*Drymarchon couperi*), American bullfrog (*Lithobates catesbeianus*), Mexican redknee tarantula (*Brachypelma*)	Mixed/multiple (nonfood enrichment, scents)	Mixed/multiple (paired, single)
Ogura & Matsuzawa (2012)	Japanese macaques (*Macaca fuscata*)	Symbols (thumbnails of videos)	Array
Passos et al. (2014)	Tortoises (*Chelonoidis denticulata*)	Mixed/multiple (food‐based enrichment, nonfood enrichment)	Mixed/multiple (single, array)
Perdue et al. (2014)	Capuchin monkeys (*Cebus)*, rhesus macaques (*Macaca mulatta*)	Symbols (icons representing computer tasks)	Mixed/multiple (paired, array)
Remis (2002)	Western lowland gorillas (*Gorilla gorilla gorilla*), chimpanzees (*Pan troglodytes*)	Foods	Paired
Sasson‐Yenor & Powell (2019)	Rothschild's giraffe (*Giraffa camelopardalis rothschildi)*	Mixed/multiple (food, food‐based enrichment)	Mixed/multiple (paired with duplication, presented to group)
Schwartz et al. (2016)	Capuchin monkeys (*Cebus apella*)	Food	Paired
Slocum & Morris (2022)	Black vultures (*Coragyps atratus*), turkey vulture (*Cathartes aura*)	Food	Paired
Sullivan et al. (2016)	Goldfish (*Carassius auratus*)	Nonfood (plant enrichment)	Paired
Truax & Vonk (2021)	Western lowland gorillas (*Gorilla gorilla gorilla)*	Symbols (icons representing sounds)	Mixed/multiple (paired, array)
Vonk et al. (2022)	Western lowland gorillas (*Gorilla gorilla gorilla)*	Mixed/multiple (foods, symbols/images of foods)	Paired
Woods et al. (2020)	lions (*Panthera leo*)	Mixed/multiple (scents, nonfood enrichment)	Paired

In most of these animal preference studies, some measure of choice (e.g., approach, consumption) was the main dependent variable. However, some studies took an additional step to determine if preference translated to reinforcer efficacy. Similar to findings in the human literature, most studies have shown that higher preference items served as more effective reinforcers than lower preference items, resulting in more lever presses (Dixon et al., 2016), more touchscreen touches (Hopper et al., 2019), more touches to training targets (Martin et al., 2018), more enrichment use (Fay & Miller, 2015; Fernandez & Timberlake, 2019b; Mehrkam & Dorey, 2014; Woods et al., 2020), higher engagement in husbandry training (Martin et al., 2018), or swimming against stronger currents (Sullivan et al., 2016). However, one study (Schwartz et al., 2016) showed no difference in amount of work capuchin monkeys (*Cebus apella*) were willing to perform (as measured by weight lifted) based on preference as assessed via pair‐wise presentation. Preference assessments have also been used to investigate concepts such as preference for novelty (Addessi et al., 2005), variety (Addessi, 2008; Addessi et al., 2010), work/contrafreeloading (Sasson‐Yenor & Powell, 2019), or choice (Perdue et al., 2014). Preference stability across time has also been investigated (Addessi et al., 2010; Clay et al., 2009; Hopper et al., 2019; Martin et al., 2018; Vonk et al., 2022). All these findings can be used to guide animal management practices to optimize welfare (see Broom & Johnson, 2019). These protocols also offer zoo animals choice, which is increasingly considered an important component of animal welfare (Melfi & Ward, 2020; Patterson‐Kane et al., 2008; Wickins‐Drazilova, 2006).

#### Untapped Applications of Applied Behavior Analysis Protocols

Although we have highlighted two behavioral protocols that were developed in human treatment clinics and adapted for use in the zoo (functional analysis and preference assessments), there are many other behavioral protocols that could be useful in zoo settings. For example, a veterinarian having difficulty getting an animal to consume oral medications could borrow from the applied behavior analytic literature related to blending (Mueller et al., 2004) or the use of chasers (Vaz et al., 2012; see Daly et al., 2019) to increase food consumption. A zookeeper working to train a long‐duration behavior could model their training after other applied research involving percentile schedules (e.g., Athens et al., 2007; Galbicka, 1994; Hall et al., 2009). Continued and additional bi‐directional translation from applied research with humans to applied research with zoo animals has considerable potential. However, realizing the full potential of applied behavior analysis in zoo settings will require the training of more individuals who are both well‐versed in the fundamentals of behavior analysis and who have experience in animal management, so that new behavioral protocols can be developed and implemented to maximize animal welfare (Fernandez & Timberlake, 2008; Friedman et al., 2021; Gray & Diller, 2017; Maple & Perdue, 2013; Maple & Segura, 2015; Martin, 2017).

### Single‐Case Designs and the Zoo

A final note worth making is the importance of single‐case designs in the study and improvement of the behavioral welfare of zoo animals. Single‐case methods are used to analyze how the behavior of an individual changes over time (Skinner, 1957; Skinner, 1966b). The zoo environment has a strong need for understanding how individuals change over time, given the limited number of animals often exhibited in any one enclosure, as well as the demand for determining the function of any event, such as environmental enrichment, on the individual behaviors of those zoo‐housed animals (Alligood et al., 2017; Fernandez & Timberlake, 2008; Maple & Segura, 2015). Unfortunately, a common misconception made by those working in zoos and similar applied settings is that without sufficient numbers of individuals to perform between‐subject comparisons, the only options left are case studies or observation‐only designs (Fernandez, 2022; Kazdin, 2021). However, as noted in the paragraph above and by the title of this section, single‐case designs offer quantitative, empirical solutions to optimally understand zoo animal behavior. Some of the many benefits of single‐case designs (over between‐subject designs) include (a) a focus on many data points from a few individuals (as opposed to few data points from many individuals), (b) an emphasis on inductive data collection that modifies procedures based on real‐time results (as opposed to a priori hypothesis testing), and (c) the assessment of an individual's learning repeatedly and over time (as opposed to pre‐ vs. posttest analyses; Bailey & Burch, 2017; DeRosa et al., 2021; Johnston & Pennypacker, 2010). In short, all research efforts aimed at improving the lives of individual animals are studies of *n* = 1 (Walker & Carr, 2021). Even if it were possible to produce enough subjects to run a standard between‐subject study on some welfare advancement (generally not the case, given the limitations of exhibit space size and/or species numbers within zoos), providing individualized welfare plans based on differences between the average of some group is of limited use if the animal in question does not respond in the average way. Single‐case designs allow for the empirical examination of each individual animal's important behavior contingencies to best promote welfare.

Forthman and Odgen (1992) saw the great potential for applied behavior analysis and zoo collaborations. In the three decades since their publication, applied behavior analysis has provided the scientific basis for many empirical studies that have both advanced applied science and have improved the welfare of zoo animals. Behavior analytic methodologies and their focus on the overt behaviors of individuals is ideally suited for improving the lives of zoo animals, and it is our hope that this updated review of applied behavior analysis in zoos provides a proper framework for both past efforts and future endeavors to continue making such research possible.

## References

[jaba969-bib-0001] Addessi, E. (2008). Food variety‐seeking in tufted capuchin monkeys (*Cebus apella*). Physiology & Behavior, 93(1–2), 304–309. 10.1016/j.physbeh.2007.09.001 17904595

[jaba969-bib-0002] Addessi, E. , Mancini, A. , Crescimbene, L. , Ariely, D. , & Visalberghi, E. (2010). How to spend a token? Trade‐offs between food variety and food preference in tufted capuchin monkeys (*Cebus apella*). Behavioural Processes, 83(3), 267–275. 10.1016/j.beproc.2009.12.012 20026196

[jaba969-bib-0003] Addessi, E. , Stammati, M. , Sabbatini, G. , & Visalberghi, E. (2005). How tufted capuchin monkeys (*Cebus apella*) rank monkey chow in relation to other foods. Animal Welfare, 14(3), 215–222.

[jaba969-bib-0004] Alligood, C. A. , Daneault, A. J. , Carlson, R. C. , Dillenbeck, T. , Wheaton, C. J. , & Savage, A. (2011). Development of husbandry practices for the captive breeding of Key Largo woodrats (*Neotoma floridana smalli*). Zoo Biology, 30(3), 318–327. 10.1002/zoo.20205 20853415

[jaba969-bib-0005] Alligood, C. A. , Dorey, N. R. , Mehrkam, L. R. , & Leighty, K. A. (2017). Applying behavior‐analytic methodology to the science and practice of environmental enrichment in zoos and aquariums. Zoo Biology, 36(3), 175–185. 10.1002/zoo.21368 29165867

[jaba969-bib-0006] Alligood, C. A. , Wheaton, C. J. , Forde, H. M. , Smith, K. N. , Daneault, A. J. , Carlson, R. C. , & Savage, A. (2008). Pup development and maternal behavior in captive Key Largo woodrats (*Neotoma floridana smalli*). Zoo Biology, 27(5), 394–405. 10.1002/zoo.20205 19360633

[jaba969-bib-0007] Allsop, S. E. , Dundas, S. J. , Adams, P. J. , Kreplins, T. L. , Bateman, P. W. , & Fleming. P. A. (2017). Reduced efficacy of baiting programs for invasive species: Some mechanisms and management implications. Pacific Conservation Biology, 23(3), 240–257. 10.1071/PC17006

[jaba969-bib-0008] Andrews, N. L. , & Ha, J. C. (2014). The effects of automated scatter feeders on captive grizzly bear activity budgets. Journal of Applied Animal Welfare Science, 17(2), 148–156. 10.1080/10888705.2013.856767 24467390

[jaba969-bib-0009] Antkowiak, K. , & Hayes, T. (2004). Rodent pest control through the reintroduction of an extirpated raptor species. Endangered Species Update, 21(4), 124–128.

[jaba969-bib-0010] Association of Zoos and Aquariums (2022). Species Survival Plan Programs [On‐line]. https://www.aza.org/species-survival-plan-programs

[jaba969-bib-0011] Athens, E. S. , Vollmer, T. R. , & St. Peter Pipkin, C. C. (2007). Shaping academic task engagement with percentile schedules. Journal of Applied Behavior Analysis, 40(3), 475–488. 10.1901/jaba.2007.40-475 17970261 PMC1986693

[jaba969-bib-0012] Bailey, J. S. , & Burch, M. R. (2017). Research methods in applied behavior analysis. Routledge.

[jaba969-bib-0013] Baker, K. C. , Bloomsmith, M. A. , Neu, K. , Griffis, C. , & Maloney, M. (2010). Positive reinforcement training as enrichment for singly housed rhesus macaques (*Macaca mulatta*). Animal Welfare, 19(3), 307–313. https://pubmed.ncbi.nlm.nih.gov/25960611 25960611 PMC4423822

[jaba969-bib-0014] Bashaw, M. J. (2011). Consistency of captive giraffe behavior under two different management regimes. Zoo Biology, 30(4), 371–378. 10.1002/zoo.20338 20717898

[jaba969-bib-0015] Bashaw, M. J. , Bloomsmith, M. A. , Maple, T. L. , & Bercovitch, F. B. (2007). The structure of social relationships among captive female giraffe (*Giraffa camelopardalis*). Journal of Comparative Psychology, 121(1), 46. 10.1037/0735-7036.121.1.46 17324074

[jaba969-bib-0016] Baum, W. M. (2012). Rethinking reinforcement: Allocation, induction, and contingency. Journal of the Experimental Analysis of Behavior, 97(1), 101–124. 10.1901/jeab.2012.97-101 22287807 PMC3266735

[jaba969-bib-0017] Beavers, G. A. , Iwata, B. A. , & Lerman, D. C. (2013). Thirty years of research on the functional analysis of problem behavior. Journal of Applied Behavior Analysis, 46(1), 1–21. 10.1002/jaba.30 24114081

[jaba969-bib-0018] * Bloomfield, R. C. , Gillespie, G. R. , Kerswell, K. J. , Butler, K. L. , & Hemsworth, P. H. (2015). Effect of partial covering of the visitor viewing area window on positioning and orientation of zoo orangutans: A preference test. Zoo Biology, 34(3), 223–229. 10.1002/zoo.21207 25716803

[jaba969-bib-0019] Bloomsmith, M. A. , & Lambeth, S. P. (1995). Effects of predictable versus unpredictable feeding schedules on chimpanzee behavior. Applied Animal Behaviour Science, 44(1), 65–74. 10.1016/0168-1591(95)00570-I

[jaba969-bib-0020] Bloomsmith, M. A. , Marr, M. J. , & Maple, T. L. (2007). Addressing nonhuman primate behavioral problems through the application of operant conditioning: Is the human treatment approach a useful model? Applied Animal Behaviour Science, 102(3–4), 205–222. 10.1016/j.applanim.2006.05.028

[jaba969-bib-0021] Bloomsmith, M. A. , Stone, A. M. , & Laule, G. E. (1998). Positive reinforcement training to enhance the voluntary movement of group‐housed chimpanzees within their enclosures. Zoo Biology, 17(4), 333–341. 10.1002/(SICI)1098-2361(1998)17:4<333::AID-ZOO6>3.0.CO;2-A

[jaba969-bib-0022] Brando, S. I. (2012). Animal learning and training: Implications for animal welfare. Veterinary Clinics: Exotic Animal Practice, 15(3), 387–398. 10.1016/j.cvex.2012.06.008 22998957

[jaba969-bib-0023] Breland, K. , & Breland, M. (1951). A field of applied animal psychology. American Psychologist, 6(6), 202. 10.1037/h0063451 14847139

[jaba969-bib-0024] Breland, K. , & Breland, M. (1961). The misbehavior of organisms. American Psychologist, 16(11), 681. 10.1037/h0040090

[jaba969-bib-0025] Broom, D. M. , & Johnson, K. G. (2019). Preference studies and welfare. In D. M. Broom & K. G. Johnson (Eds.), Stress and animal welfare (Vol. 19, pp. 173–191). Springer. 10.1007/978-3-030-32153-6_7

[jaba969-bib-0026] Brown, P. L. , & Jenkins, H. M. (1968). Auto‐shaping of the pigeon's key‐peck. Journal of the Experimental Analysis of Behavior, 11(1), 1–8. 10.1901/jeab.1968.11-1 5636851 PMC1338436

[jaba969-bib-0027] Browning, H. (2020). The natural behavior debate: Two conceptions of animal welfare. Journal of Applied Animal Welfare Science, 23(3), 325–337. 10.1080/10888705.2019.1672552 31559855

[jaba969-bib-0028] * Brox, B. W. , Edwards, K. , Buist, N. A. , & Macaskill, A. C. (2021). Investigating food preference in zoo‐housed meerkats. Zoo Biology, 40(6), 517–526. 10.1002/zoo.21640 34270126

[jaba969-bib-0029] Bryan, K. , Bremner‐Harrison, S. , Price, E. , & Wormell, D. (2017). The impact of exhibit type on behaviour of caged and free‐ranging tamarins. Applied Animal Behaviour Science, 193, 77–86. 10.1016/j.applanim.2017.03.013

[jaba969-bib-0030] Carey, P. W. , O'Connor, C. E. , McDonald, R. M. , & Matthews, L. R. (1997). Comparison of the attractiveness of acoustic and visual stimuli for brushtail possums. New Zealand Journal of Zoology, 24(4), 273–276. 10.1080/03014223.1997.9518124

[jaba969-bib-0031] Carlstead, K. , Brown, J. L. , & Seidensticker, J. (1993). Behavioral and adrenocortical responses to environmental changes in leopard cats (*Felis bengalensis*). Zoo Biology, 12(4), 321–331. 10.1002/zoo.1430120403

[jaba969-bib-0032] Carlstead, K. , & Seidensticker, J. (1991). Seasonal variation in stereotypic pacing in an American black bear (*Ursus americanus)* . Behavioural Processes, 25(2–3), 155–161. 10.1016/0376-6357(91)90017-T 24923974

[jaba969-bib-0033] Carlstead, K. , Seidensticker, J. , & Baldwin, R. (1991). Environmental enrichment for zoo bears. Zoo Biology, 10(1), 3–16. 10.1002/zoo.1430100103

[jaba969-bib-0034] Carr, J. E. , Nicolson, A. C. , & Higbee, T. S. (2000). Evaluation of a brief multiple‐stimulus preference assessment in a naturalistic context. Journal of Applied Behavior Analysis, 33(3), 353–357. 10.1901/jaba.2000.33-353 11051581 PMC1284262

[jaba969-bib-0035] Carter, M. , Sherwen, S. , & Webber, S. (2021). An evaluation of interactive projections as digital enrichment for orangutans. Zoo Biology, 40(2), 107–114. 10.1002/zoo.21587 33503300

[jaba969-bib-0036] Castro, M. I. , Beck, B. B. , Kleiman, D. G. , Ruiz‐Miranda, C. R. , & Rosenberger, A. L. (1998). Environmental enrichment in a reintroduction program for golden lion tamarins. In D. J. Shepherdson , J. D. Mellen , & M. Hutchins (Eds.), Second nature: Environmental enrichment for captive animals (pp. 97–128). Smithsonian Books.

[jaba969-bib-0037] Chiandetti, C. , Avella, S. , Fongaro, E. , & Cerri, F. (2016). Can clicker training facilitate conditioning in dogs? Applied Animal Behaviour Science, 184, 109–116. 10.1016/j.applanim.2016.08.006

[jaba969-bib-0038] Clapperton, B. K. , Morgan, D. K. J. , Day, T. D. , Oates, K. E. , Beath, A. M. , Cox, N. R. , & Matthews, L. R. (2014). Efficacy of bird repellents at deterring North Island robins (*Petroica australis longipes*) and tomtits (*P. macrocephala toitoi*) from baits. New Zealand Journal of Ecology, 38(1), 116–123. http://www.jstor.org/stable/24060829

[jaba969-bib-0039] Clapperton, B. K. , Day, T. D. , Morgan, D. K. J. , Huddart, F. , Cox, N. , & Matthews, L. R. (2015). Palatability and efficacy to possums and rats of pest control baits containing bird repellents. New Zealand Journal of Zoology, 42(2), 104–118. 10.1080/03014223.2015.1029496

[jaba969-bib-0040] Clay, A. W. , Bloomsmith, M. A. , Marr, M. J. , & Maple, T. L. (2009). Systematic investigation of the stability of food preferences in captive orangutans: Implications for positive reinforcement training. Journal of Applied Animal Welfare Science, 12(4), 306–313. 10.1080/10888700903163492 20183483

[jaba969-bib-0041] Clayton, M. , & Shrock, T. (2020). Making a tiger's day: Free‐operant assessment and environmental enrichment to improve the daily lives of captive Bengal tigers (*Panthera tigris tigris*). Behavior Analysis in Practice, 13(4), 883–893. 10.1007/s40617-020-00478-z 33269198 PMC7666234

[jaba969-bib-0042] Coe, J . (2004). Mixed species rotation exhibits. In 2004 ARAZPA Conference Proceedings. https://docslib.org/doc/935621/mixed-species-rotation-exhibits-2004-arazpa-conference-proceedings-australia-on-cd

[jaba969-bib-0043] Coe, J. , & Hoy, J. (2020). Choice, control and computers: Empowering wildlife in human care. Multimodal Technologies and Interaction, 4(4), 92–109. 10.3390/mti4040092

[jaba969-bib-0044] Cote, C. A. , Thompson, R. H. , Hanley, G. P. , & McKerchar, P. M. (2007). Teacher report and direct assessment of preferences for identifying reinforcers for young children. Journal of Applied Behavior Analysis, 40(1), 157–166. 10.1901/jaba.2007.177-05 17471799 PMC1868818

[jaba969-bib-0045] * Cox, M. , Gaglione, E. , Prowten, P. , & Noonan, M. (1996). Food preferences communicated via symbol discrimination by a California sea lion (*Zalophus californianus*). Aquatic Mammals, 22(1), 3–10.

[jaba969-bib-0046] D'Cruze, N. , Khan, S. , Carder, G. , Megson, D. , Coulthard, E. , Norrey, J. , & Groves, G. (2019). A global review of animal–visitor interactions in modern zoos and aquariums and their implications for wild animal welfare. Animals, 9(6), 332–352. 10.3390/ani9060332 31181769 PMC6617332

[jaba969-bib-0047] Dadone, L. I. , Schilz, A. , Friedman, S. G. , Bredahl, J. , Foxworth, S. , & Chastain, B. (2016). Training giraffe (*Giraffa camelopardalis reticulata*) for front foot radiographs and hoof care. Zoo Biology, 35(3), 228–236. 10.1002/zoo.21279 26991999

[jaba969-bib-0048] Daly, M. B. , Clayton, A. M. , Ruone, S. , Mitchell, J. , Dinh, C. , Holder, A. , Jolly, J. , Garcia‐Lerma, J. G. , & Weed, J. L. (2019). Training rhesus macaques to take daily oral antiretroviral therapy for preclinical evaluation of HIV prevention and treatment strategies. Plos One, 14(11), e0225146. 10.1371/journal.pone.0225146 31730629 PMC6857902

[jaba969-bib-0049] Davey, G. (2007). Visitors' effects on the welfare of animals in the zoo: A review. Journal of Applied Animal Welfare Science, 10(2), 169–183. 10.1080/10888700701313595 17559323

[jaba969-bib-0050] DeLeon, I. G. , & Iwata, B. A. (1996). Evaluation of a multiple‐stimulus presentation format for assessing reinforcer preferences. Journal of Applied Behavior Analysis, 29(4), 519–533. 10.1901/jaba.1996.29-519 8995834 PMC1284008

[jaba969-bib-0051] DeRosa, N. M. , Sullivan, W. E. , Roane, H. S. , Craig, A. R. , & Kadey, H. J. (2021). Single‐case experimental design. In W. W. Fisher , C. C. Piazza , & H. S. Roane (Eds). Handbook of applied behavior analysis (2nd ed., pp. 155–171). Guilford Press.

[jaba969-bib-0052] Dixon, M. R. , Daar, J. H. , Gunnarsson, K. , Johnson, M. L. , & Shayter, A. M. (2016). Stimulus preference and reinforcement effects of the Madagascar hissing cockroach (*Gromphordahina portentosa*): A case of reverse translational research. The Psychological Record, 66(1), 41–51. 10.1007/s40732-015-0149-9

[jaba969-bib-0053] Domjan, M. (1983). Biological constraints on instrumental and classical conditioning: Implications for general process theory. Psychology of Learning & Motivation, 17, 215–277. 10.1016/S0079-7421(08)60100-0

[jaba969-bib-0054] Domjan, M. , Blesbois, E. , & Williams, J. (1998). The adaptive significance of sexual conditioning: Pavlovian control of sperm release. Psychological Science, 9(5), 411–415. 10.1111/1467-9280.00077

[jaba969-bib-0055] Dorey, N. R. , Blandina, A. , & Udell, M. A. (2020). Clicker training does not enhance learning in mixed‐breed shelter puppies (*Canis familiaris*). Journal of Veterinary Behavior, 39, 57–63. 10.1016/j.jveb.2020.07.005

[jaba969-bib-0056] Dorey, N. R. , & Cox, D. J. (2018). Function matters: A review of terminological differences in applied and basic clicker training research. PeerJ, 6, e5621. 10.7717/peerj.5621 30258718 PMC6151118

[jaba969-bib-0057] Dorey, N. R. , Mehrkam, L. R. , & Tacey, J. (2015). A method to assess relative preference for training and environmental enrichment in captive wolves (*Canis lupus* and *Canis lupus arctos*). Zoo Biology, 34(6), 513–517. 10.1002/zoo.21239 26274933

[jaba969-bib-0058] Dorey, N. R. , Rosales‐Ruiz, J. , Smith, R. , & Lovelace, B. (2009). Functional analysis and treatment of self‐injury in a captive olive baboon. Journal of Applied Behavior Analysis, 42(4), 785–794. 10.1901/jaba.2009.42-785 20514183 PMC2790931

[jaba969-bib-0059] Dorey, N. R. , Tobias, J. S. , Udell, M. A. , & Wynne, C. D. (2012). Decreasing dog problem behavior with functional analysis: Linking diagnoses to treatment. Journal of Veterinary Behavior, 7(5), 276–282. 10.1016/j.jveb.2011.10.002

[jaba969-bib-0060] Edwards, T. L. , & Poling, A. (2011). Animal research in the *Journal of Applied Behavior Analysi*s. Journal of Applied Behavior Analysis, 44(2), 409–412. 10.1901/jaba.2011.44-409 21709802 PMC3120082

[jaba969-bib-0061] Egger, M. D. , & Miller, N. E. (1962). Secondary reinforcement in rats as a function of information value and reliability of the stimulus. Journal of Experimental Psychology, 64(2), 97. 10.1037/h0040364 13889429

[jaba969-bib-0062] English, M. , Kaplan, G. , & Rogers, L. J. (2014). Is painting by elephants in zoos as enriching as we are led to believe? PeerJ, 2, e471. 10.7717/peerj.471 25071994 PMC4103097

[jaba969-bib-0063] Eskelinen, H. C. , Winship, K. A. , & Borger‐Turner, J. L. (2015). Sex, age, and individual differences in bottlenose dolphins (*Tursiops truncatus*) in response to environmental enrichment. Animal Behavior and Cognition, 2(3), 241–253. 10.12966/abc.08.04.2015

[jaba969-bib-0064] Fa, J. E. , Funk, S. M. , & O'Connell, D. (2011). Zoo conservation biology. Cambridge University Press.

[jaba969-bib-0065] Fantino, E. (1969). Choice and rate of reinforcement. Journal of the Experimental Analysis of Behavior, 12(5), 723–730. 10.1901/jeab.1969.12-723 16811396 PMC1338674

[jaba969-bib-0066] Fantino, E. , & Romanowich, P. (2007). The effect of conditioned reinforcement rate on choice: A review. Journal of the Experimental Analysis of Behavior, 87(3), 409–421. 10.1901/jeab.2007.44-06 17575906 PMC1868584

[jaba969-bib-0067] Farmer, H. L. , Plowman, A. B. , & Leaver, L. A. (2011). Role of vocalisations and social housing in breeding in captive howler monkeys (*Alouatta caraya*). Applied Animal Behaviour Science, 134(3–4), 177–183. 10.1016/j.applanim.2011.07.005

[jaba969-bib-0068] Farmer‐Dougan, V. (2014). Functional analysis of aggression in a black‐and‐white ruffed lemur (*Varecia variegata variegata*). Journal of Applied Animal Welfare Science, 17(3), 282–293. 10.1080/10888705.2014.917029 24911429

[jaba969-bib-0069] Fay, C. , & Miller, L. J. (2015). Utilizing scents as environmental enrichment: Preference assessment and application with Rothschild giraffe. Animal Behavior and Cognition, 2(3), 285–291. 10.12966/abc.08.07.2015

[jaba969-bib-0070] Fernandez, E. J. (2001). Click or treat: A trick or two in the zoo. American Animal Trainer Magazine, 2(2), 41–44.

[jaba969-bib-0071] Fernandez, E. J. (2017). The empirical zoo in the 21st century: The professor in the zoo, Maple, T. L Red Leaf Press, Tequesta, FL, 2016. pp. 360. Zoo Biology, 36(2), 170–171. 10.1002/zoo.21356

[jaba969-bib-0072] Fernandez, E. J. (2020). Training petting zoo sheep to act like petting zoo sheep: An empirical evaluation of response‐independent schedules and shaping with negative reinforcement. Animals, 10(7), 1122–1135. 10.3390/ani10071122 32630257 PMC7401582

[jaba969-bib-0073] Fernandez, E. J. (2021). Appetitive search behaviors and stereotypies in polar bears (*Ursus maritimus*). Behavioural Processes, 182, 104299. 10.1016/j.beproc.2020.104299 33358744

[jaba969-bib-0074] Fernandez, E. J. (2022). Training as enrichment: A critical review. Animal Welfare, 31(1), 1–12. 10.7120/09627286.31.1.001

[jaba969-bib-0075] Fernandez, E. J. , & Chiew, S. J. (2021). Animal‐visitor interactions: Effects, experiences, and welfare. Animal Behavior and Cognition, 8(4), 462–467. 10.26451/abc.08.04.13.2021

[jaba969-bib-0076] Fernandez, E. J. , & Dorey, N. R. (2021). An examination of shaping with an African crested porcupine (*Hystrix cristata*). Journal of Applied Animal Welfare Science, 24(4), 372–378. 10.1080/10888705.2020.1753191 32309999

[jaba969-bib-0077] Fernandez, E. J. , Dorey, N. , & Rosales‐Ruiz, J. (2004). A two‐choice preference assessment with five cotton‐top tamarins (*Saguinus oedipus*). Journal of Applied Animal Welfare Science, 7(3), 163–169. 10.1207/s15327604jaws0703_2 15498723

[jaba969-bib-0078] Fernandez, E. , & Harvey, E. (2021). Enclosure use as a measure of behavioral welfare in zoo‐housed African wild dogs (*Lycaon pictus*). Journal of Zoo and Aquarium Research, 9(2), 88–93. 10.19227/jzar.v9i2.526

[jaba969-bib-0079] Fernandez, E. J. , Kinley, R. C. , & Timberlake, W. (2019). Training penguins to interact with enrichment devices for lasting effects. Zoo Biology, 38(6), 481–489. 10.1002/zoo.21510 31355481

[jaba969-bib-0080] Fernandez, E. J. , & Martin, A. L. (2021). Animal training, environment enrichment, and animal welfare: A history of behavior analysis in zoos. Journal of Zoological and Botanical Gardens, 2(4), 531–543. 10.3390/jzbg2040038

[jaba969-bib-0081] Fernandez, E. J. , Myers, M. , & Hawkes, N. C. (2021). The effects of live feeding on swimming activity and exhibit use in zoo Humboldt penguins (*Spheniscus humboldti*). Journal of Zoological and Botanical Gardens, 2(1), 88–100. 10.3390/jzbg2010007

[jaba969-bib-0082] Fernandez, E. J. , Ramirez, M. , & Hawkes, N. C. (2020). Activity and pool use in relation to temperature and water changes in zoo hippopotamuses (*Hippopotamus amphibious*). Animals, 10(6), 1022. 10.3390/ani10061022 32545610 PMC7341244

[jaba969-bib-0083] Fernandez, E. J. , & Rosales‐Ruiz, J. (2021). A comparison of fixed‐time food schedules and shaping involving a clicker for halter behavior in a petting zoo goat. The Psychological Record, 71(3), 487–491. 10.1007/s40732-020-00420-3

[jaba969-bib-0084] Fernandez, E. J. , Tamborski, M. A. , Pickens, S. R. , & Timberlake, W. (2009). Animal–visitor interactions in the modern zoo: Conflicts and interventions. Applied Animal Behaviour Science, 120(1–2), 1–8. 10.1016/j.applanim.2009.06.002

[jaba969-bib-0085] Fernandez, E. J. , & Timberlake, W. (2008). Mutual benefits of research collaborations between zoos and academic institutions. Zoo Biology, 27(6), 470–487. 10.1002/zoo.20215 19360641

[jaba969-bib-0086] Fernandez, E. J. , & Timberlake, W. (2019a). Foraging devices as enrichment in captive walruses (*Odobenus rosmarus*). Behavioural Processes, 168, 103943. 10.1016/j.beproc.2019.103943 31479700

[jaba969-bib-0087] Fernandez, E. J. , & Timberlake, W. (2019b). Selecting and testing environmental enrichment in lemurs. Frontiers in Psychology, 10, 2119. 10.3389/fpsyg.2019.02119 31572280 PMC6753196

[jaba969-bib-0088] Fernandez, E. J. , Upchurch, B. , & Hawkes, N. C. (2021). Public feeding interactions as enrichment for three zoo‐housed elephants. Animals, 11(6), 1689. 10.3390/ani11061689 34204020 PMC8229577

[jaba969-bib-0089] Fisher, W. , Piazza, C. C. , Bowman, L. G. , Hagopian, L. P. , Owens, J. C. , & Slevin, I. (1992). A comparison of two approaches for identifying reinforcers for persons with severe and profound disabilities. Journal of Applied Behavior Analysis, 25(2), 491–498. 10.1901/jaba.1992.25-491 1634435 PMC1279726

[jaba969-bib-0090] Fisher, W. W. , Groff, R. A. , & Roane, H. S. (2021). Applied behavior analysis: History, philosophy, principles, and basic methods. In W. W. Fisher , C. C. Piazza , & H. S. Roane (Eds.), Handbook of applied behavior analysis (2nd ed., pp. 3–12). Guilford Publications.

[jaba969-bib-0091] Fisher, W. W. , Piazza, C. C. , & Roane, H. S. (Eds.). (2021). Handbook of applied behavior analysis *(* *2nd ed*. *)*. Guilford Publications.

[jaba969-bib-0092] Forthman, D. L. , & Ogden, J. J. (1992). The role of applied behavior analysis in zoo management: Today and tomorrow. Journal of Applied Behavior Analysis, 25(3), 647–652. 10.1901/jaba.1992.25-647 16795790 PMC1279745

[jaba969-bib-0093] Forthman‐Quick, D. L. (1984). An integrative approach to environmental engineering in zoos. Zoo Biology, 3(1), 65–77. 10.1002/zoo.1430030107

[jaba969-bib-0094] Franklin, A. N. , Martin, A. L. , Perlman, J. E. , & Bloomsmith, M. A. (2022). Functional analysis and treatment of disruptive behavior in a rhesus macaque. Journal of Applied Animal Welfare Science, 25(3), 287–296. 10.1080/10888705.2021.1931868 34056962 PMC9836391

[jaba969-bib-0095] Fraser, D. (2008). Understanding animal welfare. Acta Veterinaria Scandinavica, 50(1), 1–7. 10.1186/1751-0147-50-S1-S1 19049678 PMC4235121

[jaba969-bib-0096] Friedman, S. G. , Stringfield, C. E. , & Desmarchelier, M. R. (2021). Animal behavior and learning: Support from applied behavior analysis. Veterinary Clinics: Exotic Animal Practice, 24(1), 1–16. 10.1016/j.cvex.2020.08.002 33189245

[jaba969-bib-0097] Gaalema, D. E. (2013). Sexual conditioning in the dyeing poison dart frog (*Dendrobates tinctorius*). International Journal of Comparative Psychology, 26(1), 5–18.

[jaba969-bib-0098] Gaalema, D. E. , Perdue, B. M. , & Kelling, A. S. (2011). Food preference, keeper ratings, and reinforcer effectiveness in exotic animals: The value of systematic testing. Journal of Applied Animal Welfare Science, 14, 33–41. 10.1080/10888705.2011.527602 21191846

[jaba969-bib-0099] Galbicka, G. (1994). Shaping in the 21st century: Moving percentile schedules into applied settings. Journal of Applied Behavior Analysis, 27(4), 739–760. 10.1901/jaba.1994.27-739 16795849 PMC1297861

[jaba969-bib-0100] Gilchrist, R. J. , Gunter, L. M. , Anderson, S. F. , & Wynne, C. D. (2021). The click is not the trick: The efficacy of clickers and other reinforcement methods in training naïve dogs to perform new tasks. PeerJ, 9, e10881. 10.7717/peerj.10881 33665026 PMC7906040

[jaba969-bib-0101] Godinez, A. M. , & Fernandez, E. J. (2019). What is the zoo experience? How zoos impact a visitor's behaviors, perceptions, and conservation efforts. Frontiers in Psychology, 10, 1746. 10.3389/fpsyg.2019.01746 31417469 PMC6682629

[jaba969-bib-0102] Grandin, T. , Rooney, M. B. , Phillips, M. , Cambre, R. C. , Irlbeck, N. A. , & Graffam, W. (1995). Conditioning of nyala (*Tragelaphus angasi*) to blood sampling in a crate with positive reinforcement. Zoo Biology, 14(3), 261–273. 10.1002/zoo.1430140307

[jaba969-bib-0103] Gray, J. M. , & Diller, J. W. (2017). Evaluating the work of applied animal behaviorists as applied behavior analysis. Behavior Analysis: Research and Practice, 17(1), 33–41. 10.1037/bar0000041

[jaba969-bib-0104] Green, C. W. , Reid, D. H. , White, L. K. , Halford, R. C. , Brittain, D. P. , & Gardner, S. M. (1988). Identifying reinforcers for persons with profound handicaps: Staff opinion versus systematic assessment of preferences. Journal of Applied Behavior Analysis, 21(1), 31–43. 10.1901/jaba.1988.21-31 2967274 PMC1286091

[jaba969-bib-0105] Hall, N. J. , Protopopova, A. , & Wynne, C. D. (2015). The role of environmental and owner‐provided consequences in canine stereotypy and compulsive behavior. Journal of Veterinary Behavior, 10(1), 24–35. 10.1016/j.jveb.2014.10.005

[jaba969-bib-0106] Hall, S. S. , Maynes, N. P. , & Reiss, A. L. (2009). Using percentile schedules to increase eye contact in children with fragile X syndrome. Journal of Applied Behavior Analysis, 42(1), 171–176. 10.1901/jaba.2009.42-171 19721738 PMC2649838

[jaba969-bib-0107] Hancocks, D. (1980). Bringing nature into the zoo: Inexpensive solutions for zoo environments. International Journal for the Study of Animal Problems, 1(3), 170–177. https://www.wellbeingintlstudiesrepository.org/ijsap/vol1/iss3/7

[jaba969-bib-0108] Hanley, G. P. , Iwata, B. A. , & McCord, B. E. (2003). Functional analysis of problem behavior: A review. Journal of Applied Behavior Analysis, 36(2), 147–185. 10.1901/jaba.2003.36-147 12858983 PMC1284431

[jaba969-bib-0109] Hediger, H. (1950). Wild animals in captivity. Butterworths Scientific Publications.

[jaba969-bib-0110] Hediger, H. (1955). Studies of the psychology and behavior of captive animals in zoos and circuses. Butterworths Scientific Publications.

[jaba969-bib-0111] Herrnstein, R. J. (1977). The evolution of behaviorism. American Psychologist, 32(8), 593–603. 10.1037/0003-066X.32.8.593

[jaba969-bib-0112] Hollis, K. L. , Pharr, V. L. , Dumas, M. J. , Britton, G. B. , & Field, J. (1997). Classical conditioning provides paternity advantage for territorial male blue gouramis (*Trichogaster trichopterus*). Journal of Comparative Psychology, 111(3), 219–225. 10.1037/0735-7036.111.3.219

[jaba969-bib-0113] Hopper, L. M. , Egelkamp, C. L. , Fidino, M. , & Ross, S. R. (2019). An assessment of touchscreens for testing primate food preferences and valuations. Behavior Research Methods, 51(2), 639–650. 10.3758/s13428-018-1065-0 29949070

[jaba969-bib-0114] Hosey, G. R. (2000). Zoo animals and their human audiences: What is the visitor effect? Animal Welfare, 9(4), 343–357.

[jaba969-bib-0115] Hosey, G. , Birke, L. , Shaw, W. S. , & Melfi, V. (2018). Measuring the strength of human–animal bonds in zoos. Anthrozoös, 31(3), 273–281. 10.1080/08927936.2018.1455448

[jaba969-bib-0116] * Huskisson, S. M. , Egelkamp, C. L. , Jacobson, S. L. , Ross, S. R. , & Hopper, L. M. (2021). Primates' food preferences predict their food choices even under uncertain conditions. Animal Behavior and Cognition, 8(1), 69–96. 10.26451/abc.08.01.06.2021

[jaba969-bib-0117] * Huskisson, S. M. , Jacobson, S. L. , Egelkamp, C. L. , Ross, S. R. , & Hopper, L. M. (2020). Using a touchscreen paradigm to evaluate food preferences and response to novel photographic stimuli of food in three primate species (*Gorilla gorilla gorilla*, *Pan troglodytes*, and *Macaca fuscata*). International Journal of Primatology, 41(1), 5–23. 10.1007/s10764-020-00131-0

[jaba969-bib-0118] Hutchins, M. (2006). Variation in nature: Its implications for zoo elephant management. Zoo Biology, 25(3), 161–171. 10.1002/zoo.20087

[jaba969-bib-0119] Hutchins, M. , Hancocks, D. , & Crockett, C. (1984). Naturalistic solutions to the behavioral problems of captive animals. Der Zoologische Garten, 54, 28–42.

[jaba969-bib-0120] Inglis, I. R. , Forkman, B. , & Lazarus, J. (1997). Free food or earned food? A review and fuzzy model of contrafreeloading. Animal Behaviour, 53(6), 1171–1191. 10.1006/anbe.1996.0320 9236014

[jaba969-bib-0121] Iwata, B. A. , Dorsey, M. F. , Slifer, K. J. , Bauman, K. E. , & Richman, G. S. (1994). Toward a functional analysis of self‐injury. Journal of Applied Behavior Analysis, 27(2), 197–209. (Reprinted from *Analysis and Intervention in Developmental Disabilities*, 2, 3–20, 1982) 10.1901/jaba.1994.27-197 8063622 PMC1297798

[jaba969-bib-0122] Jensen, G. D. (1963). Preference for bar pressing over “freeloading” as a function of number of rewarded presses. Journal of Experimental Psychology, 65(5), 451. 10.1037/h0049174 13957621

[jaba969-bib-0123] Johnston, J. M. , & Pennypacker, H. S. (2010). Strategies and tactics of behavioral research. Routledge.

[jaba969-bib-0124] Kazdin, A. E. (2021). Single‐case experimental designs: Characteristics, changes, and challenges. Journal of the Experimental Analysis of Behavior, 115(1), 56–85. 10.1002/jeab.638 33205436

[jaba969-bib-0125] Kelleher, R. T. , & Gollub, L. R. (1962). A review of positive conditioned reinforcement. Journal of the Experimental Analysis of Behavior, 5(S4), 543–597. 10.1901/jeab.1962.5-s543 14031747 PMC1404082

[jaba969-bib-0126] Killeen, P. R. (2019). Timberlake's theories dissolve anomalies. Behavioural Processes, 166, 103894. 10.1016/j.beproc.2019.103894 31278969

[jaba969-bib-0127] Kleiman, D. G. , Beck, B. B. , Dietz, J. M. , Dietz, L. A. , Ballou, J. D. , & Coimbra‐Filho, A. F. (1986). Conservation program for the golden lion tamarin. In K. Benirschke (Ed.), Primates: The road to self‐sustaining populations (pp. 959–979). Springer‐Verlag.

[jaba969-bib-0128] Kreger, M. D. , & Mench, J. A. (1995). Visitor‐animal interactions at the zoo. Anthrozoös, 8(3), 143–158. 10.2752/089279395787156301

[jaba969-bib-0129] Kyngdon, D. J. , Minot, E. O. , & Stafford, K. J. (2003). Behavioural responses of captive common dolphins *Delphinus delphis* to a ‘Swim‐with‐Dolphin’ programme. Applied Animal Behaviour Science, 81(2), 163–170. 10.1016/S0168-1591(02)00255-1

[jaba969-bib-0130] Lasky, M. , Campbell, J. , Osborne, J. A. , Ivory, E. L. , Lasky, J. , & Kendall, C. J. (2021). Increasing browse and social complexity can improve zoo elephant welfare. Zoo Biology, 40(1), 9–19. 10.1002/zoo.21575 33043537

[jaba969-bib-0131] Lattal, K. A. , & Fernandez, E. J. (2022). Grounding applied animal behavior practices in the experimental analysis of behavior. Journal of the Experimental Analysis of Behavior, 118(2), 186–207. 10.1002/jeab.789 36043528

[jaba969-bib-0132] Lauderdale, L. , Samuelson, M. , & Xitco, M. (2021). Modern applications of operant conditioning through the training of a beaching behaviour with bottlenose dolphins (*Tursiops truncatus*). Journal of Zoo and Aquarium Research, 9(2), 81–87. 10.19227/jzar.v9i2.519

[jaba969-bib-0133] Learmonth, M. J. , Chiew, S. J. , Godinez, A. , & Fernandez, E. J. (2021). Animal‐visitor interactions and the visitor experience: Visitor behaviors, attitudes, perceptions, and learning in the modern zoo. Animal Behavior and Cognition, 8, 632–649. 10.26451/abc.08.04.13.2021

[jaba969-bib-0134] Lee, M. S. , Yu, C. T. , Martin, T. L. , & Martin, G. L. (2010). On the relation between reinforcer efficacy and preference. Journal of Applied Behavior Analysis, 43(1), 95–100. 10.1901/jaba.2010.43-95 20808498 PMC2831456

[jaba969-bib-0135] Lukas, K. E. , Marr, M. J. , & Maple, T. L. (1998). Teaching operant conditioning at the zoo. Teaching of Psychology, 25(2), 112–116. 10.1207/s15328023top2502_7

[jaba969-bib-0136] Maple, T. L. (2007). Toward a science of welfare for animals in the zoo. Journal of Applied Animal Welfare Science, 10(1), 63–70. 10.1080/10888700701277659 17484680

[jaba969-bib-0137] Maple, T. L. (2008). Empirical zoo: Opportunities and challenges to a scientific zoo biology. Zoo Biology, 27(6), 431–435. 10.1002/zoo.20214 19360637

[jaba969-bib-0138] Maple, T. L. (2016). The rise and fall of animal behavior labs: The future of comparative psychology. Animal Behavior and Cognition, 3(3), 131–143. 10.12966/abc.02.08.2016

[jaba969-bib-0139] Maple, T. (2017). Professor in the zoo: Designing the future for wildlife in human care. Red Leaf Press.

[jaba969-bib-0140] Maple, T. L. (2021). The practice of management: The ascent of women as scholars and leaders in the field of zoo biology. The Psychologist‐Manager Journal, 24(2), 97–114. 10.1037/mgr0000114

[jaba969-bib-0141] Maple, T. L. , & Perdue, B. M. (2013). Behavior analysis and training. In T. L. Maple & B. M. Perdue (Eds.), Zoo animal welfare (pp. 119–137). Springer.

[jaba969-bib-0142] Maple, T. L. , & Segura, V. D. (2015). Advancing behavior analysis in zoos and aquariums. The Behavior Analyst, 38(1), 77–91. 10.1007/s40614-014-0018-x 27540508 PMC4883490

[jaba969-bib-0143] Markowitz, H. (1978). Engineering environments for behavioral opportunities in the zoo. The Behavior Analyst, 1(1), 34–47. 10.1007/BF03392371 22477954 PMC2741797

[jaba969-bib-0144] Markowitz, H. (1982). Behavioral enrichment in the zoo. Van Nostrand Reinhold.

[jaba969-bib-0145] Markowitz, H. , & LaForse, S. (1987). Artificial prey as behavioral enrichment devices for felines. Applied Animal Behaviour Science, 18, 31–43. 10.1016/0168-1591(87)90252-8

[jaba969-bib-0146] Markowitz, H. , Schmidt, M. J. , & Moody, A. (1978). Behavioural engineering and animal health in the zoo. International Zoo Yearbook, 18(1), 190–194. 10.1111/j.1748-1090.1978.tb00256.x

[jaba969-bib-0147] Martin, A. L. (2017). The primatologist as a behavioral engineer. American Journal of Primatology, 79(1), e22500. 10.1002/ajp.22500 PMC7427841 26539749

[jaba969-bib-0148] Martin, A. L. , Bloomsmith, M. A. , Kelley, M. E. , Marr, M. J. , & Maple, T. L. (2011). Functional analysis and treatment of human‐directed undesirable behavior exhibited by a captive chimpanzee (*Pan troglodytes*). Journal of Applied Behavior Analysis, 44(1), 139–143. 10.1901/jaba.2011.44-139 21541106 PMC3050472

[jaba969-bib-0149] Martin, A. L. , Franklin, A. N. , Perlman, J. E. , & Bloomsmith, M. A. (2018). Systematic assessment of food item preference and reinforcer effectiveness: Enhancements in training laboratory‐housed rhesus macaques. Behavioural Processes, 157, 445–452. 10.1016/j.beproc.2018.07.002 30003936 PMC6240383

[jaba969-bib-0150] McGowan, R. T. , Robbins, C. T. , Alldredge, J. R. , & Newberry, R. C. (2010). Contrafreeloading in grizzly bears: Implications for captive foraging enrichment. Zoo Biology, 29(4), 484–502. 10.1002/zoo.20282 19816856

[jaba969-bib-0151] McPhee, M. E. (2003). Effects of captivity on response to a novel environment in the Oldfield mouse (*Peromyscus polionotus subgriseus*). International Journal of Comparative Psychology, 16(2), 85–94. 10.46867/C45C7H

[jaba969-bib-0152] Mehrkam, L. R. , & Dorey, N. R. (2014). Is preference a predictor of enrichment efficacy in Galapagos tortoises (*Chelonoidis nigra*)? Zoo Biology, 33(4), 275–284. 10.1002/zoo.21151 25065472

[jaba969-bib-0153] Mehrkam, L. R. , & Dorey, N. R. (2015). Preference assessments in the zoo: Keeper and staff predictions of enrichment preferences across species. Zoo Biology, 34(5), 418–430. 10.1002/zoo.21227 26179195

[jaba969-bib-0154] Mehrkam, L. R. , Perez, B. C. , Self, V. N. , Vollmer, T. R. , & Dorey, N. R. (2020). Functional analysis and operant treatment of food guarding in a pet dog. Journal of Applied Behavior Analysis, 53(4), 2139–2150. 10.1002/jaba.720 32390171

[jaba969-bib-0155] Melfi, V. A. , & Ward, S. J. (2020). Welfare implications of zoo animal training. In V. A. Melfi , N. R. Dorey , & S. J. Ward (Eds.), Zoo animal learning and training (pp. 271–288). John Wiley & Sons Ltd. 10.1002/9781118968543.ch11

[jaba969-bib-0156] Miller, B. , Biggins, D. , Vargas, A. , Hutchins, M. , Hanebury, L. , Godbey, J. , Anderson, S. , Wemmer, C. , & Oldemeier, J. (1998). The captive environment and reintroduction: The black‐footed ferret as a case study with comments on other taxa. In D. J. Shepherdson , J. D. Mellen , & M. Hutchins (Eds.), Second nature: Environmental enrichment for captive animals (pp. 97–112). Smithsonian Press.

[jaba969-bib-0157] Morgan, D. R. (1990). Behavioral‐response of brushtail possums, *Trichosurus‐vulpecula*, to baits used in pest‐control. Australian Wildlife Research, 17(6), 601–613. 10.1071/WR9900601

[jaba969-bib-0158] Morris, D. (1964). The response of animals to a restricted environment. Symposia of the Zoological Society of London, 13, 99–118.

[jaba969-bib-0159] Morris, E. K. , Lazo, J. F. , & Smith, N. G. (2004). Whether, when, and why Skinner published on biological participation in behavior. The Behavior Analyst, 27(2), 153–169. 10.1007/BF03393176 22478425 PMC2755402

[jaba969-bib-0160] Morris, K. L. , & Slocum, S. K. (2019). Functional analysis and treatment of self‐injurious feather plucking in a black vulture (*Coragyps atratus*). Journal of Applied Behavior Analysis, 52(4), 918–927. 10.1002/jaba.639 31523815

[jaba969-bib-0161] Mueller, M. M. , Piazza, C. C. , Patel, M. R. , Kelley, M. E. , & Pruett, A. (2004). Increasing variety of foods consumed by blending nonpreferred foods into preferred foods. Journal of Applied Behavior Analysis, 37(2), 159–170. 10.1901/jaba.2004.37-159 15293635 PMC1284491

[jaba969-bib-0162] Neuringer, A. J. (1969). Animals respond for food in the presence of free food. Science, 166(3903), 399–401. 10.1126/science.166.3903.399 5812041

[jaba969-bib-0163] Newberry, R. C. (1995). Environmental enrichment: Increasing the biological relevance of captive environments. Applied Animal Behaviour Science, 44(2–4), 229–243. 10.1016/0168-1591(95)00616-Z

[jaba969-bib-0164] * Ogura, T. , & Matsuzawa, T. (2012). Video preference assessment and behavioral management of single‐caged Japanese macaques (*Macaca fuscata*) by movie presentation. Journal of Applied Animal Welfare Science, 15(2), 101–112. 10.1080/10888705.2012.624887 22458872

[jaba969-bib-0165] Owen, M. A. , Swaisgood, R. R. , Czekala, N. M. , & Lindburg, D. G. (2005). Enclosure choice and well‐being in giant pandas: Is it all about control? Zoo Biology, 24(5), 475–481. 10.1002/zoo.20064

[jaba969-bib-0166] Pace, G. M. , Ivancic, M. T. , Edwards, G. L. , Iwata, B. A. , & Page, T. J. (1985). Assessment of stimulus preference and reinforcer value with profoundly retarded individuals. Journal of Applied Behavior Analysis, 18(3), 249–255. 10.1901/jaba.1985.18-249 4044458 PMC1308015

[jaba969-bib-0167] Padrell, M. , Amici, F. , Córdoba, M. P. , Giberga, A. , Broekman, A. , Almagro, S. , & Llorente, M. (2021). Artificial termite‐fishing tasks as enrichment for sanctuary‐housed chimpanzees: Behavioral effects and impact on welfare. Animals, 11(10), 2941. 10.3390/ani11102941 34679962 PMC8532803

[jaba969-bib-0168] * Passos, L. F. , Mello, H. E. S. , & Young, R. J. (2014). Enriching tortoises: Assessing color preference. Journal of Applied Animal Welfare Science, 17(3), 274–281. 10.1080/10888705.2014.917556 24911428

[jaba969-bib-0169] Patterson‐Kane, E. G. , Pittman, M. , & Pajor, E. A. (2008). Operant animal welfare: Productive approaches and persistent difficulties. Animal Welfare, 17(2), 139–148.

[jaba969-bib-0170] Perdue, B. M. , Evans, T. A. , Washburn, D. A. , Rumbaugh, D. M. , & Beran, M. J. (2014). Do monkeys choose to choose? Learning & Behavior, 42, 164–175. 10.3758/s13420-014-0135-0 24567075 PMC4196680

[jaba969-bib-0171] Pfaller‐Sadovsky, N. , Arnott, G. , & Hurtado‐Parrado, C. (2019). Using principles from applied behaviour analysis to address an undesired behaviour: Functional analysis and treatment of jumping up in companion dogs. Animals, 9(12), 1091. 10.3390/ani9121091 31817670 PMC6940775

[jaba969-bib-0172] Pfaller‐Sadovsky, N. , Hurtado‐Parrado, C. , Cardillo, D. , Medina, L. G. , & Friedman, S. G. (2020). What's in a click? The efficacy of conditioned reinforcement in applied animal training: A systematic review and meta‐analysis. Animals, 10(10), 1757. 10.3390/ani10101757 32998242 PMC7600771

[jaba969-bib-0173] Phillips, M. , Grandin, T. , Graffam, W. , Irlbeck, N. A. , & Cambre, R. C. (1998). Crate conditioning of bongo (*Tragelaphus eurycerus*) for veterinary and husbandry procedures at the Denver Zoological Gardens. Zoo Biology, 17(1), 25–32. 10.1002/(SICI)1098-2361(1998)17:1<25::AID-ZOO3>3.0.CO;2-C

[jaba969-bib-0174] Piazza, C. C. , Fisher, W. W. , Hagopian, L. P. , Bowman, L. G. , & Toole, L. (1996). Using a choice assessment to predict reinforcer effectiveness. Journal of Applied Behavior Analysis, 29(1), 1–9. 10.1901/jaba.1996.29-1 8881340 PMC1279869

[jaba969-bib-0175] Pierce, W. D. , & Cheney, C. D. (2013). Behavior analysis and learning. Psychology Press.

[jaba969-bib-0176] Price, E. C. , Wormell, D. , Brayshaw, M. , Furrer, S. , Heer, T. , & Steinmetz, H. W. (2012). Managing free‐ranging callitrichids in zoos. International Zoo Yearbook, 46(1), 123–136. 10.1111/j.1748-1090.2012.00167.x

[jaba969-bib-0177] Pryor, K. (1984). Don't shoot the dog:The new art of teaching and training. Bantam Books.

[jaba969-bib-0178] * Remis, M. J. (2002). Food preferences among captive Western gorillas (*Gorilla gorilla gorilla*) and chimpanzees (*Pan troglodytes*). International Journal of Primatology, 23(2), 231–249. 10.1023/A:1013837426426

[jaba969-bib-0179] Ritzler, C. P. , Lukas, K. E. , Bernstein‐Kurtycz, L. M. , & Koester, D. C. (2021). The effects of choice‐based design and management on the behavior and space use of zoo‐housed amur tigers (*Panthera tigris altaica*). Journal of Applied Animal Welfare Science, 6, 1‐14. 10.1080/10888705.2021.1958684 34353192

[jaba969-bib-0180] Rose, N. A. , & Parsons, E. C. M. (2019). The case against marine mammals in captivity (5th ed.). Animal Welfare Institute and World Animal Protection.

[jaba969-bib-0181] Rowden, J. (2001). Behavior of captive Bulwer's wattled pheasants, *Lophura bulweri* (Galliformes: Phasianidae). Zoo Biology, 20(1), 15–25. 10.1002/zoo.1002 11319777

[jaba969-bib-0182] Ruiz‐Miranda, C. R. , de Morais Jr, M. M. , Dietz, L. A. , Rocha Alexandre, B. , Martins, A. F. , Ferraz, L. P. , Mickelberg, J. , Hankerson, S. J. , & Dietz, J. M. (2019). Estimating population sizes to evaluate progress in conservation of endangered golden lion tamarins (*Leontopithecus rosalia*). Plos One, 14(6), e0216664. 10.1371/journal.pone.0216664 31166940 PMC6550383

[jaba969-bib-0183] Saini, V. , Retzlaff, B. , Roane, H. S. , & Piazza, C. C. (2021). Identifying and enhancing the effectiveness of positive reinforcement. In W. W. Fisher , C. C. Piazza , & H. S. Roane (Eds.) Handbook of applied behavior analysis (2nd ed., pp. 175–192). Guilford Publications.

[jaba969-bib-0184] Salmeron, M. C. , Payne, S. W. , & Hegr, A. B. (2021). Functional analysis and treatment of feline aggression in an animal shelter. Behavior Analysis: Research and Practice, 21(2), 128–139. 10.1037/bar0000185

[jaba969-bib-0185] Sanders, K. , & Fernandez, E. J. (2020). Behavioral implications of enrichment for golden lion tamarins: A tool for ex situ conservation. Journal of Applied Animal Welfare Science, 25(3), 214–223. 10.1080/10888705.2020.1809413 32841087

[jaba969-bib-0186] Sasson‐Yenor, J. , & Powell, D. M. (2019). Assessment of contrafreeloading preferences in giraffe (*Giraffa camelopardalis*). Zoo Biology, 38(5), 414–423. 10.1002/zoo.21513 31432564

[jaba969-bib-0187] Saudargas, R. A. , & Drummer, L. C. (1996). Single subject (small N) research designs and zoo research. Zoo Biology, 15(2), 173–181. 10.1002/(SICI)1098-2361(1996)15:2<173::AID-ZOO7>3.0.CO;2-8

[jaba969-bib-0188] Savastano, G. , Hanson, A. , & McCann, C. **(** 2003). The development of an operant conditioning training program for New World primates at the Bronx Zoo. Journal of Applied Animal Welfare Science, 6(3), 247–261. 10.1207/S15327604JAWS0603_09 14612272

[jaba969-bib-0189] Schmid, J. , Heistermann, M. , Gansloßer, U. , & Hodges, J. K. (2001). Introduction of foreign female Asian elephants (*Elephas maximus*) into an existing group: Behavioural reactions and changes in cortisol levels. Animal Welfare, 10(4), 357–372.

[jaba969-bib-0190] Schwartz, L. P. , Silberberg, A. , Casey, A. H. , Paukner, A. , & Suomi, S. J. (2016). Scaling reward value with demand curves versus preference tests. Animal Cognition, 19(3), 631–641. 10.1007/s10071-016-0967-4 26908005 PMC4826314

[jaba969-bib-0191] Shepherdson, D. J. , Carlstead, K. , Mellen, J. D. , & Seidensticker, J. (1993). The influence of food presentation on the behavior of small cats in confined environments. Zoo Biology, 12(2), 203–216. 10.1002/zoo.1430120206

[jaba969-bib-0192] Sherwen, S. L. , & Hemsworth, P. H. (2019). The visitor effect on zoo animals: Implications and opportunities for zoo animal welfare. Animals, 9(6), 366. 10.3390/ani9060366 31212968 PMC6617010

[jaba969-bib-0193] Shettleworth, S. J. (1993). Varieties of learning and memory in animals. Journal of Experimental Psychology: Animal Behavior Processes, 19, 5–14. 10.1037/0097-7403.19.1.5 8418217

[jaba969-bib-0194] Shyne, A. (2006). Meta‐analytic review of the effects of enrichment on stereotypic behavior in zoo mammals. Zoo Biology, 25(4), 317–337. 10.1002/zoo.20091

[jaba969-bib-0195] Siciliano‐Martina, L. , & Martina, J. P. (2018). Stress and social behaviors of maternally deprived captive giraffes (*Giraffa camelopardalis*). Zoo Biology, 37(2), 80–89. 10.1002/zoo.21405 29527718

[jaba969-bib-0196] Skinner, B. F. (1938). The behavior of organisms. Appleton‐Century‐Crofts.

[jaba969-bib-0197] Skinner, B. F. (1951). How to teach animals. Scientific American, 185(6), 26–29. https://www.jstor.org/stable/24950550#metadata_info_tab_contents:~:text=StableURL-,https://www.jstor.org/stable/24950550,-RemoteAccessURL

[jaba969-bib-0198] Skinner, B. F. (1953). Science and human behavior. The Free Press.

[jaba969-bib-0199] Skinner, B. F. (1957). The experimental analysis of behavior. American Scientist, 45(4), 343–371. https://www.jstor.org/stable/27826953

[jaba969-bib-0200] Skinner, B. F. (1966a). The phylogeny and ontogeny of behavior: Contingencies of reinforcement throw light on contingencies of survival in the evolution of behavior. Science, 153(3741), 1205–1213. 10.1126/science.153.3741.1205 5918710

[jaba969-bib-0201] Skinner, B. F. (1966b). What is the experimental analysis of behavior? Journal of the Experimental Analysis of Behavior, 9(3), 213–218. 10.1901/jeab.1966.9-213 16811287 PMC1338181

[jaba969-bib-0202] Skinner, B. (1984). Selection by consequences. Behavioral and Brain Sciences, 7(4), 477–481. doi:10.1017/S0140525X0002673X

[jaba969-bib-0203] * Slocum, S. K. , & Morris, K. L. (2022). Assessing preference in a paired‐stimulus arrangement with captive vultures (*Aegypius monachus*). Journal of Applied Animal Welfare Science, 25(4), 362–367. 10.1080/10888705.2020.1857253 33305965

[jaba969-bib-0204] Stoinski, T. S. , Beck, B. B. , Bloomsmith, M. A. , & Maple, T. L. (2003). A behavioral comparison of captive‐born, reintroduced golden lion tamarins and their wild‐born offspring. Behaviour, 140(2), 137–160. http://www.jstor.org/stable/4536018

[jaba969-bib-0205] Stoinski, T. S. , Beck, B. B. , Bowman, M. , & Lehnhardt, J. (1997). The gateway zoo program: A recent initiative in golden lion tamarin zoo introductions. In J. Wallis (Ed.), Primate conservation: The role of zoological parks (pp. 113–129). American Society of Primatologists.

[jaba969-bib-0206] Sullivan, M. , Lawrence, C. , & Blache, D. (2016). Why did the fish cross the tank? Objectively measuring the value of enrichment for captive fish. Applied Animal Behaviour Science, 174, 181–188. 10.1016/j.applanim.2015.10.011

[jaba969-bib-0207] Swaisgood, R. R. , & Shepherdson, D. J. (2005). Scientific approaches to enrichment and stereotypies in zoo animals: What's been done and where should we go next? Zoo Biology, 24(6), 499–518. 10.1002/zoo.20066

[jaba969-bib-0208] Timberlake, W. (1984). A temporal limit on the effect of future food on current performance in an analogue of foraging and welfare. Journal of the Experimental Analysis of Behavior, 41(2), 117–124. 10.1901/jeab.1984.41-117 16812361 PMC1348025

[jaba969-bib-0209] Timberlake, W. (1993). Behavior systems and reinforcement: An integrative approach. Journal of the Experimental Analysis of Behavior, 60(1), 105–128. 10.1901/jeab.1993.60-105 8354963 PMC1322149

[jaba969-bib-0210] Timberlake, W. (2004). Is the operant contingency enough for a science of purposive behavior? Behavior and Philosophy, 197–229. https://www.jstor.org/stable/27759478

[jaba969-bib-0211] Timberlake, W. , & Lucas, G. A. (1989). Behavior systems and learning: From misbehavior to general principles In S. B. Klein & R. R. Mowrer (Eds.), Contemporary learning theories: Instrumental conditioning theory and the impact of biological constraints on learning (pp. 237–275). Lawrence Erlbaum Associates.

[jaba969-bib-0212] Trone, M. , Kuczaj, S. , & Solangi, M. (2005). Does participation in Dolphin–Human Interaction Programs affect bottlenose dolphin behaviour? Applied Animal Behaviour Science, 93(3–4), 363–374. 10.1016/j.applanim.2005.01.003

[jaba969-bib-0213] Troxell‐Smith, S. M. , Whelani, C. J. , Magle, S. B. , & Brown, S. (2017). Zoo foraging ecology: Development and assessment of a welfare tool for captive animals. Animal Welfare, 26(3), 265–275. 10.7120/09627286.26.3.265

[jaba969-bib-0214] * Truax, J. , & Vonk, J. (2021). Silence is golden: Auditory preferences in zoo‐housed gorillas. Journal of Applied Animal Welfare Science, 1–16. Advance online publication. 10.1080/10888705.2021.1968400 34428085

[jaba969-bib-0215] Vasconcellos, A. da S. , Adania, C. H. , & Ades, C. (2012). Contrafreeloading in maned wolves: Implications for their management and welfare. Applied Animal Behaviour Science, 140(1‐2), 85–91. 10.1016/j.applanim.2012.04.012

[jaba969-bib-0216] Vaz, P. C. M. , Piazza, C. C. , Stewart, V. , Volkert, V. M. , Groff, R. A. , & Patel, M. R. (2012). Using a chaser to decrease packing in children with feeding disorders. Journal of Applied Behavior Analysis, 45(1), 97–105. 10.1901/jaba.2012.45-97 22403452 PMC3297356

[jaba969-bib-0217] Veeder, C. L. , Bloomsmith, M. A. , McMillan, J. L. , Perlman, J. E. , & Martin, A. L. (2009). Positive reinforcement training to enhance the voluntary movement of group‐housed sooty mangabeys (*Cercocebus atys atys*). Journal of the American Association for Laboratory Animal Science, 48(2), 192–195. 19383217 PMC2679662

[jaba969-bib-0218] Vonk, J. , Truax, J. , & McGuire, M. C. (2022). A food for all seasons: Stability of food preferences in gorillas across testing methods and seasons. Animals, 12(6), 685. 10.3390/ani12060685 35327082 PMC8944577

[jaba969-bib-0219] Wagman, J. D. , Lukas, K. E. , Dennis, P. M. , Willis, M. A. , Carroscia, J. , Gindlesperger, C. , & Schook, M. W. (2018). A work‐for‐food enrichment program increases exploration and decreases stereotypies in four species of bears. Zoo Biology, 37(1), 3–15. 10.1002/zoo.21391 29315790

[jaba969-bib-0220] Walker, S. G. , & Carr, J. E. (2021). Generality of findings from single‐case designs: It's not all about the “N.” Behavior Analysis in Practice, 14(4), 991–995. 10.1007/s40617-020-00547-3 34868812 PMC8586328

[jaba969-bib-0221] Ward, S. J. , & Melfi, V. (2015). Keeper‐animal interactions: Differences between the behaviour of zoo animals affect stockmanship. PloS One, 10(10), e0140237. 10.1371/journal.pone.0140237 26509670 PMC4624973

[jaba969-bib-0222] Wheaton, C. J. , Alligood, C. , Pearson, M. , Gleeson, T. , & Savage, A. (2013). First report of alloparental care in the Key Largo woodrat (*Neotoma floridana smalli*). Journal of Ethology, 31(3), 331–334. 10.1007/s10164-013-0378-9

[jaba969-bib-0223] Wickins‐Drazilova, D. (2006). Zoo animal welfare. Journal of Agricultural and Environmental Ethics, 19(1), 27–36. 10.1007/s10806-005-4380-2

[jaba969-bib-0224] Williams, B. A. (1994). Conditioned reinforcement: Experimental and theoretical issues. The Behavior Analyst, 17(2), 261–285. 10.1007/BF03392675 22478192 PMC2733461

[jaba969-bib-0225] Williams, D. R. , & Williams, H. (1969). Auto‐maintenance in the pigeon: Sustained pecking despite contingent non‐reinforcement. Journal of the Experimental Analysis of Behavior, 12(4), 511–520. 10.1901/jeab.1969.12-511 16811370 PMC1338642

[jaba969-bib-0226] Wilson, M. L. , Perdue, B. M. , Bloomsmith, M. A. , & Maple, T. L. (2015). Rates of reinforcement and measures of compliance in free and protected contact elephant management systems. Zoo Biology, 34(5), 431–437. 10.1002/zoo.21229 26179311

[jaba969-bib-0227] Winslow, T. , Payne, S. W. , & Massoudi, K. A. (2018). Functional analysis and treatment of problem behavior in 3 animal shelter dogs. Journal of Veterinary Behavior, 26, 27–37. 10.1016/j.jveb.2018.04.004

[jaba969-bib-0228] Woods, J. M. , Lane, E. K. , & Miller, L. J. (2020). Preference assessments as a tool to evaluate environmental enrichment. Zoo Biology, 39(6), 382–390. 10.1002/zoo.21566 32813291

[jaba969-bib-0229] Yerkes, R. M. (1925). Almost human. Century.

[jaba969-bib-0230] Zhang, Z. , Gao, L. , & Zhang, X. (2022). Environmental enrichment increases aquatic animal welfare: A systematic review and meta‐analysis. Reviews in Aquaculture, 14(3), 1120‐1135. 10.1111/raq.12641

